# Evolution and separation of actinobacterial pyranose and *C*-glycoside-3-oxidases

**DOI:** 10.1128/aem.01676-23

**Published:** 2024-01-05

**Authors:** Anja Kostelac, André Taborda, Lígia O. Martins, Dietmar Haltrich

**Affiliations:** 1 Department of Food Science and Technology, BOKU—University of Natural Resources and Life Sciences, Vienna, Austria; 2 Doctoral Programme BioToP—Biomolecular Technology of Proteins, BOKU—University of Natural Resources and Life Sciences, Vienna, Austria; 3 Instituto de Tecnologia Química e Biológica António Xavier, Universidade NOVA de Lisboa, Oeiras, Portugal; University of Milano-Bicocca, Milan, Italy

**Keywords:** pyranose oxidase, FAD-dependent *C*-glycoside-3-oxidase, substrate specificity, sequence space, ancestral reconstruction

## Abstract

FAD-dependent pyranose oxidase (POx) and *C*-glycoside-3-oxidase (CGOx) are both members of the glucose-methanol-choline superfamily of oxidoreductases and belong to the same sequence space. Pyranose oxidases had been studied for their oxidation of monosaccharides such as D-glucose, but recently, a bacterial *C*-glycoside-3-oxidase that is phylogenetically related to POx and that reacts with *C*-glycosides such as carminic acid, mangiferin or puerarin has been described. Since these actinobacterial CGOx enzymes belong to the same sequence space as bacterial POx, they must have evolved from the same ancestor. Here, we performed a phylogenetic analysis of actinobacterial sequences and resurrected seven ancestral enzymes of the POx/CGOx sequence space to study the evolutionary trajectory of substrate preferences for monosaccharides and *C*-glycosides. Clade I, with its dimeric member POx from *Kitasatospora aureofaciens*, shows strict preference for monosaccharides (D-glucose and D-xylose) and does not react with any of the glycosides tested. No extant member of clade II has been studied to date. The two extant members of clades III and IV, monomeric POx/CGOx from *Pseudoarthrobacter siccitolerans* and *Streptomyces canus*, oxidized both monosaccharides as well as various *C*-glycosides (homoorientin, isovitexin, mangiferin, and puerarin). Steady-state kinetic parameters of several clades III and IV ancestral enzymes indicate that the generalist ancestor N35 slowly evolved to present-day enzymes with a much higher preference for *C*-glycosides than monosaccharides. Based on structural predictions of ancestors, we hypothesize that the strict specificity of bacterial clade I POx (and also fungal POx) is the result of oligomerization, which in turn results from the evolution of protein segments that were shown to be important for oligomerization, the arm, and the head domain.

**IMPORTANCE:**

*C*-Glycosides often form active compounds in various plants. Breakage of the C-C bond in these glycosides to release the aglycone is challenging and proceeds via a two-step reaction, the oxidation of the sugar and subsequent cleavage of the C-C bond. Recently, an enzyme from a soil bacterium, FAD-dependent *C*-glycoside-3-oxidase (CGOx), was shown to catalyze the initial oxidation reaction. Here, we show that CGOx belongs to the same sequence space as pyranose oxidase (POx), and that an actinobacterial ancestor of the POx/CGOx family evolved into four clades, two of which show a high preference for *C*-glycosides.

## INTRODUCTION

Pyranose oxidase (pyranose 2-oxidase, glucose 2-oxidase; POx, EC 1.1.3.10, pyranose:oxygen 2-oxidoreductase) is a member of the glucose-methanol-choline (GMC) superfamily of oxidoreductases, oxidizing various sugars at their C-2 (and sometimes also C-3) position in the presence of oxygen (1, 2). Since its discovery in 1968 it has been studied mainly from various fungal sources [*Aspergillus oryzae*, *Aspergillus nidulans*, *Irpex lacteus*, *Lyophyllum shimeji*, *Peniophora gigantea*, *Peniophora* sp., *Phanerochaete chrysosporium*, *Phlebiopsis gigantea*, *Trametes multicolor* (*Tm*POx), and *Tricholoma matsutake*] (3). Fungal POx is thought to be involved in lignocellulose degradation. The distribution of POx in the fungal kingdom is somewhat peculiar compared to other enzyme members of the GMC superfamily. In general, it is only found in relatively few fungal species but still throughout most of the fungal kingdom, and it is rarely found in two closely related fungal species (2). It was hypothesized that the *pox* gene had been introduced into fungi via horizontal gene transfer from bacteria and thus, the functions of POx might have been redundant in a number of fungal organisms leading to its subsequent loss, or its rare occurrence across the fungal kingdom could stem from several independent late gene transfer events from bacteria. Recently, POx was also studied from bacterial sources [*Pseudoarthrobacter siccitolerans*, formerly *Arthrobacter siccitolerans* (*Ps*POx), *Streptomyces canus* (*Sc*POx), and *Kitasatospora aureofaciens* (*Ka*POx)] (4–6). Bacterial POx, or more precisely *Sc*POx and *Ps*POx, show characteristics distinct from fungal POx. Both are monomeric enzymes with a molecular mass of approx. 55–60 kDa, while fungal POx is typically homotetrameric (four subunits of ~65 kDa). *Ka*POx is positioned between the bacterial and canonical fungal POx as a homodimer (two subunits of 61 kDa each). In both *Sc*POx and *Ps*POx, the FAD is non-covalently attached, whereas it is tethered to a His in *Ka*POx as well as in fungal POx.

In 2021, Kumano et al. reported the characterization of a new type of enzyme, an FAD-dependent *C*-glycoside-3-oxidase (CGOx, EC 1.1.3.50) from *Microbacterium* 5-2b (CarA), *Microbacterium trichothecenolyticum* (*Mt*CarA), and *Arthrobacter globiformis* (*Ag*CarA), and also solved its structure (7). These enzymes were reportedly inactive on glucose but oxidized the glucose moiety of *C*-glycosides and, to a lesser extent, the glucose part of *O*-glycosides at the C-3 position. CarA, *Mt*CarA, and *Ag*CarA show activity on *C*-glycosides such as carminic acid, mangiferin, homoorientin, or isovitexin, among other glycosides (7). These glycosides are natural components often associated with various plant materials. *C*-glycosides are metabolized by enzymatic complexes that, in a first step, oxidize the carbohydrate part of the substrate, and then, in a second step, cleave the C-C bond between the oxidized sugar and the aglycone (8). Two types of *C*-glycoside-metabolizing enzymes catalysing the first step have been described: NAD(H)-dependent oxidoreductases (from intestinal organisms) (9) and oxygen-dependent enzymes such as CGOx (from soil microorganisms) (8). Our groups showed recently that both *Sc*POx and *Ps*POx can efficiently oxidize *C*-glycosides such as mangiferin and puerarin while also showing (low) activity on glucose and xylose (6). In fact, *Ps*POx has been termed a *C*-glycoside-3-oxidase based on these recent studies (10). The analysis of *Ps*POx X-ray crystal structures in complex with glucose and mangiferin, combined with mutagenesis and molecular dynamics simulations, revealed distinctive features in the active site that favor catalytically competent conformational states suitable for recognition, stabilization, and oxidation of the glucose moiety of the *C*-glycoside mangiferin (10). Since many members of this sequence space had been described as pyranose oxidases and since the *C*-glycoside-oxidizing enzymes oxidize a sugar in its pyranose form, we will be using the term pyranose oxidase for these enzymes here for consistency reasons.

Ancestral sequence reconstruction is a probabilistic-based approach to infer protein sequences that might have appeared in the past by using the sequence space of present-day proteins and a phylogenetic tree (11, 12). These resurrected proteins usually possess additional unique properties such as higher thermostability, improved solubility, or substrate promiscuity compared to their successors (13). A number of bacterial and mammalian proteins and enzymes have been studied to date by ancestral reconstruction not only for protein engineering, but also to elucidate structure-function relationships or their evolution (14–17). Bearing in mind the common sequence space of POx and CGOx (6, 7), we aimed to elucidate the underlying evolutionary significance and study how the activity for glycosides and monosaccharides evolved over time. Based on a multiple sequence alignment and a phylogenetic tree, we explored the diversity and functional variety of this bacterial sequence space by comprehensively characterizing seven different ancestral enzymes and compared their properties to extant bacterial and fungal POx. Here, we only focused on sequences of actinobacterial (Actinomycetota) origin since all hitherto characterized bacterial enzymes of the POx family (*Sc*POx, *Ka*POx, CarA, and *Ps*POx) are from species of this phylum.

## RESULTS

### Common ancestor reconstruction

**Fig 1:**
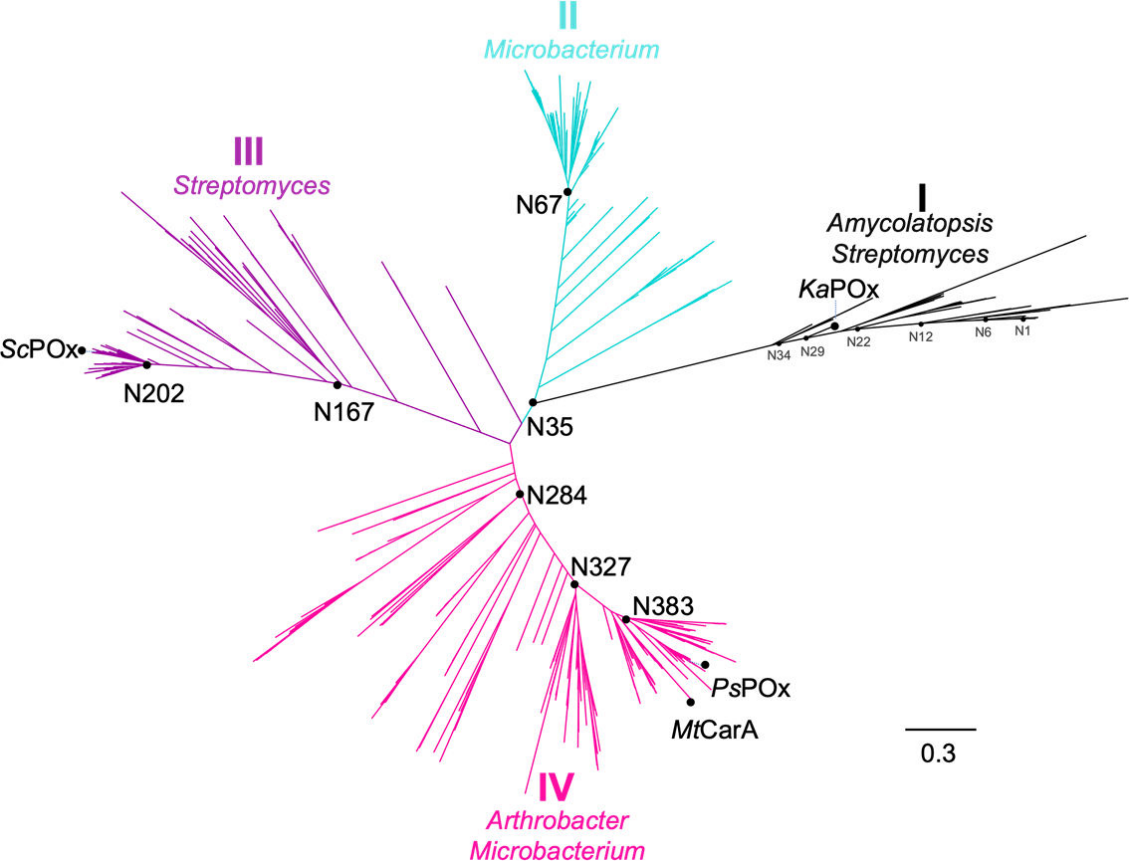
Reconstructed phylogeny of the POx and CGOx sequence space restricted to actinobacterial sequences by GRASP. Different clades are colored differently*—Amycolatopsis* and *Streptomyces* (clade I) in black, *Microbacterium* (clade II) in cyan, *Streptomyces* (clade III) in purple, and *Arthrobacter* and *Microbacterium* (clade IV) in pink. N35 is the common ancestor of all ancestral nodes included in this tree. The additional nodes N34, N29, N22, N12, N6, and N1 were also used in this study to better understand clade I based on structural investigation. The present-day enzymes *Ps*POx, *Mt*CarA, *Sc*POx, and *Ka*POx, which were previously characterized in detail, are labeled as well (4–6). No fungal POx sequences are included in the sequence space. The bar represents the phylogenetic distance as amino acid substitution per site.

Data sets for ancestral analysis were collected using several amino acid sequences that belong to the POx sequence space (5) as seeds. After curation and selection of the data, as described in the Materials and Methods section, the data set consisted of 469 sequences. The final reconstructed phylogenetic tree exhibited a pronounced topology with four different clades (Fig. 1). Most of the sequences within one clade originate from the same genera*—Amycolatopsis* and *Streptomyces* (clade I), *Microbacterium* (clade II), *Streptomyces* (clade III), and *Arthrobacter*, and *Microbacterium* (clade IV). The maximum likelihood phylogenetic tree calculated by RAxML with bootstrap values is shown in Fig. S1. Based on their position in the phylogeny, we then selected seven different ancestors for expression and further characterization. We selected node N35 as the common ancestor of all ancestral nodes within the tree, node N67 to get a better understanding of clade II, nodes N167 and N202 for clade III, and nodes N284, N327, and N383 for a closer characterization of clade IV. Posterior probabilities for each position in the primary sequence of the selected nodes confirmed the trend already known for calculation of ancestral sequences: the oldest node, in our case N35, showed the highest number of positions (110 out of 551) with ambiguous probability and thus had the lowest total posterior probability (83%). In addition, the closer to present-day sequences the calculated ancestor got, the higher the number of unambiguous places was, and thus the higher the number of total posterior probability (Table S1). The posterior probability distribution of all ancestors throughout their primary sequence is shown in Fig. S2.

### Expression of ancestral *pox* sequences and general characterization

Expression of the seven ancestral *pox* sequences was done on a 5-mL scale using two different *Escherichia coli* expression hosts [BL21(DE3) and T7(pGro7)] as well as two different induction strategies (IPTG at 30°C or lactose at 18°C), and the best-performing combination of host and induction, showing the highest activity with D-glucose and DCIP as electron acceptor, was chosen for further studies without further optimisation (Table S2). After purification of the ancestors by metal affinity chromatography, the yields varied between 0.1 and 13 mg POx protein per 1 L of liquid culture. SDS-PAGE of ancestral enzyme preparations after purification is shown the Fig. S3. Significant fractions of the purified proteins were not fully loaded with FAD as shown by spectrophotometric analysis and were hence reconstituted by incubation with free FAD after the purification step. UV/Vis spectra of the enzymes after reconstitution with FAD are shown in Fig. S4, while absorption maxima and associated extinction coefficients are summarized in Table S3. When measuring activities, obtained values were corrected for the active fraction, i.e., the enzyme fraction containing the FAD cofactor. The thermostability of ancestral enzymes was measured by the ThermoFAD assay, and the resulting thermal transition temperatures *T_m_*, indicating unfolding and release of FAD, ranged from 48 to 62°C (Table S2). It had been noted previously that ancestral proteins tend to show increased thermostabilities (13–16), which we however did not observe for most ancestors when compared to the monomeric extant enzymes *Sc*POx and *Ps*POx, except for N167 and N327, showing considerably higher *T_m_* values. Often expressability is improved for ancestral genes as well (13–16), which we also did not see for our ancestors (Table S2). All ancestral enzymes but N35 and N67 showed a monomeric state at pH 7.5. Based on size exclusion chromatography, N67 was present as a dimer and the results for N35 were ambiguous, indicating both trimeric and pentameric states, which could be the result of nonspecific protein-protein interactions. Size exclusion chromatograms of some selected ancestors are shown in Fig. S5.

### Reactivity with glycosides varies in different POx clades

**Fig 2:**
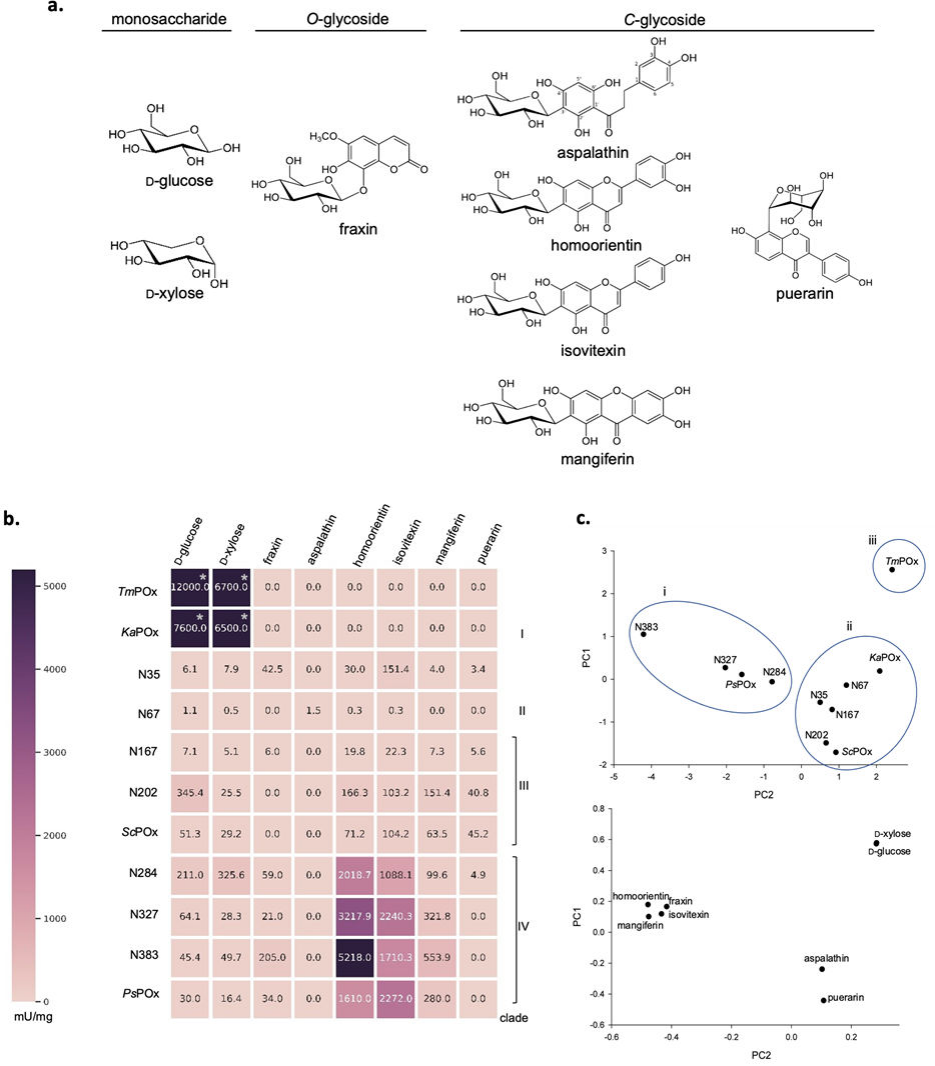
(**a**) Structures of monosaccharide as well as *C*- and *O*-glycoside substrates that were oxidized by at least one enzyme during the initial screening. (**b**) Heatmap showing specific activities (in mU/mg) of the seven POx ancestors in addition to the characterized fungal *Tm*POx and bacterial *Ka*POx, *Sc*POx, and *Ps*POx enzymes. Specific activities are shown for using 200 mM of the monosaccharide substrates and 0.2 mM of the glycoside substrates (final concentration in the assay), except for *Tm*POx and *Ka*POx, the data of which were taken from previous publications (labeled with *) (1, 5). (**c**) Principal component analysis. Upper plot (the score plot): the score plot is a graph, in which scores of the second principal component against scores of the first principal component are plotted. The groups of enzymes are numbered according to the following: (i) N393-N327-*Ps*POx-N284, (ii) *Sc*POx-N202-N167-N35-N67-*Ka*POx, and (iii) *Tm*POx. Lower plot (the loading plot); the loading plot is a graph in which coefficients of each variable for the first component against coefficients for the second component are plotted. Difference between substrates that affect the grouping of the enzymes on the score plot can be observed.

The purified ancestral enzymes were initially screened for activity with monosaccharides (D-glucose and D-xylose), 6-*C*-glycosides (aspalathin, carminic acid, homoorientin, isovitexin, and mangiferin), one 8-*C*-glycoside (puerarin), *O*-glycosides (fraxin, naringin, salicin, and rutin), and one *S*-glycoside (sinigrin). In addition, the extant proteins *Ps*POx and *Sc*POx were tested for activity with these different substrates as well, whereas *Ka*POx and *Tm*POx were only tested with *C*- and *O*-glycosides since their reactivity with the monosaccharides had been studied in detail before. This activity screening was performed using the DCIP assay (and hence dehydrogenase activity) for the POx ancestors N35, N167, N202, N284, N327, and N383 as well as for *Ps*POx and *Sc*POx, since this resulted in less background noise in the measurements. For N67, *Ka*POx, and *Tm*POx, we followed the oxidase activity using the AmplexRed- or ABTS-coupled assay, as N67 showed only negligible dehydrogenase activity, and data for the extant enzymes *Ka*POx and *Tm*POx were also reported for these assays. Activity of at least some of the POx proteins was detected for the monosaccharides D-glucose and D-xylose, the *C*-glycosides aspalathin, homoorientin, isovitexin, mangiferin, puerarin, and the *O*-glycoside fraxin (Fig. 2a). At the same time, the other substrates tested such as carminic acid, naringin, salicin, rutin, and sinigrin were not oxidized. Figure 2b summarizes and compares the specific activity data obtained for ancestors and present-day enzymes under screening conditions. All enzymes showed activity with D-glucose and D-xylose, albeit to a largely varying extent with significantly higher activities found for *Ka*POx and *Tm*POx. These two enzymes also showed no activity with any glycoside, confirming that members of the bacterial POx clade I and fungal enzymes evolved toward pronounced activity for only monosaccharides. All other enzymes showed activity with homoorientin and isovitexin. Besides that, N35 showed activity with fraxin, mangiferin and puerarin, N67 with aspalathin, N202 and *Sc*POx with mangiferin and puerarin, N167 and N284 with fraxin, mangiferin and puerarin, N327, N383, and *Ps*POx with fraxin and mangiferin. It is interesting to note that we observed a considerable increase in activity for puerarin along the phylogenetic clade III line N35-N167-N202-*Sc*POx, as well as an increase for mangiferin and isovitexin along the phylogenetic clade IV line N35-N284-N327-N383. The oldest ancestors, N35 and N284, also showed the widest reactivity with different electron donor substrates. It seems that some activities, e.g., reactivity with the *O*-glycoside fraxin in clade III or with puerarin in clade IV, were lost in these clades during evolution, possibly indicating that these enzymes evolved toward a narrower substrate reactivity and hence to an increased specialization.

The data shown in Fig. 2b were also subjected to principal component analysis (PCA) to reduce the dimensionality of the data (Fig. 2c). The upper graph in this figure, the score plot, shows the grouping of enzymes into three subjectively chosen groups (three separate distributions in the data set): N284-N327-N383-*Ps*POx, *Sc*POx-N202-N167-N35-N67-*Ka*POx, and *Tm*POx, which to some extent reflects phylogenetic lineages depicted in Fig. 1 (phylogenetic lineages N35-N67, N35-N167-N202-*Sc*POx, and N35-N284-N327-N383-*Ps*POx). The lower graph, the loading plot, identifies the monosaccharide substrates (D-xylose and D-glucose) to have a stronger positive correlation on enzyme grouping (shown on the score plot) compared to substrates that influence grouping only weakly (aspalathin, puerarin, fraxin, homoorientin, isovitexin, and mangiferin).

### Catalytic properties indicate phylogenetically distinct substrate preference

Based on the initial screening of substrate preferences and the fact that all bacterial enzymes, except *Ka*POx, reacted with the monosaccharides D-glucose and D-xylose as well as with the glycosides homoorientin and isovitexin (Fig. 2b), we selected D-glucose as a reference monosaccharide and homoorientin as a reference *C*-glycoside for the determination of the apparent steady-state constants. Additionally, N67 was characterized for aspalathin; N35, N202, and *Sc*POx for puerarin; and N35, N284, N327, N383, and *Ps*POx for fraxin. Because of the very low yield of N167 after purification, this ancestor was not further characterized.

**Fig 3:**
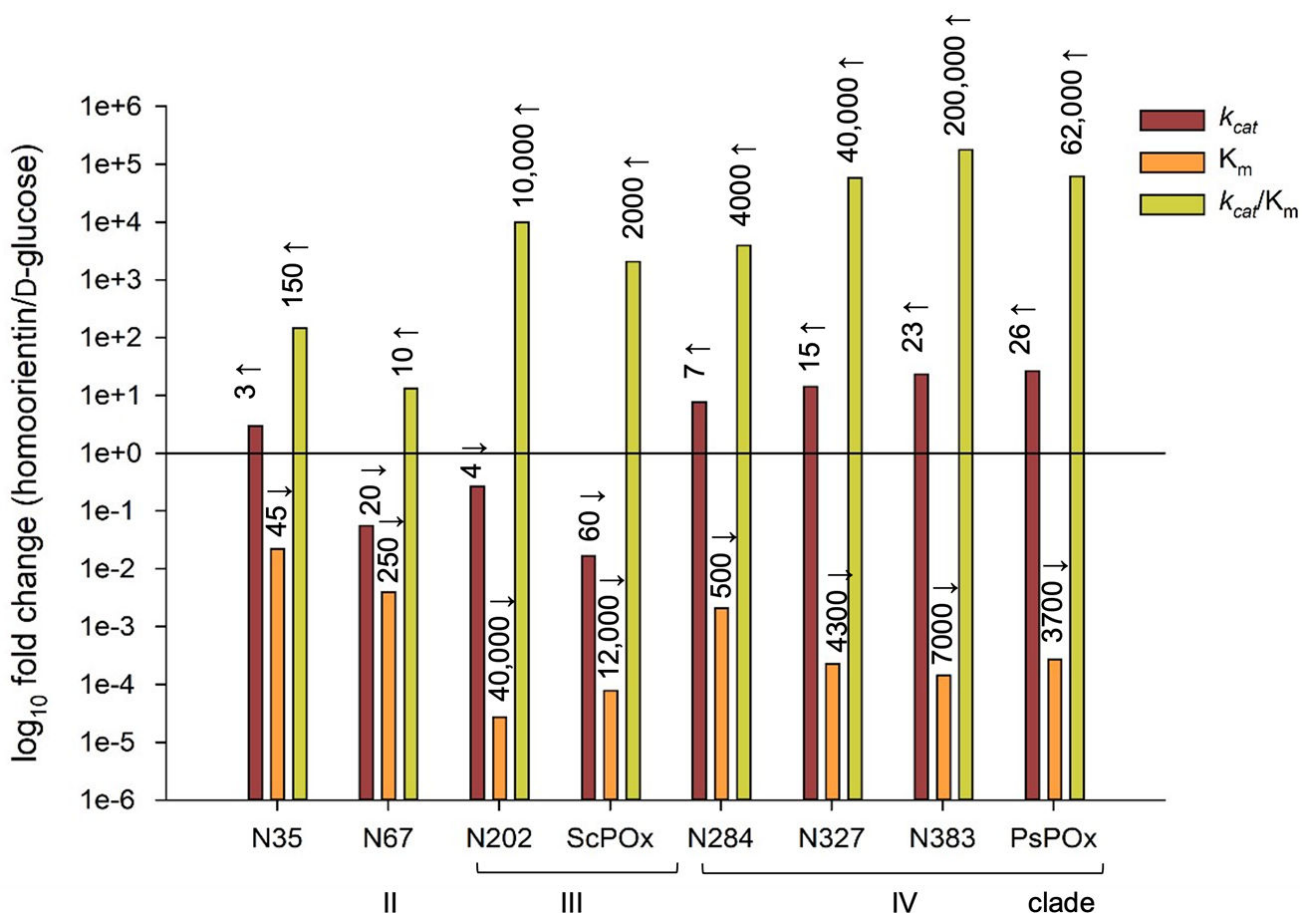
Relative change of the ratio of the steady-state kinetic constants for the *C*-glycoside homoorientin and D-glucose for N35, N67, N202, *Sc*POx, N284, N327, N383, and *Ps*POx. An increase or a decrease in this ratio is depicted by arrows (the sign ↑ represents a higher and the sign ↓ a lower value than 1e + 0).

The steady-state kinetic parameters *k_cat_*, *K*_*m*_, and *k_cat_*/*K*_*m*_ are summarised in Table 1. The fungal enzyme *Tm*POx and the bacterial clade I enzyme *Ka*POx clearly show the highest catalytic efficiency *k_cat_*/*K*_*m*_ with D-glucose. Apart from these two, the oldest ancestor N35 showed the highest catalytic efficiency based on its low Michaels constant *K*_*m*_. Furthermore, the catalytic constants for D-glucose showed a very clear tendency along the evolutionary line of ancestors. The catalytic efficiency decreased stepwise along the phylogenetic line N35-N284-N327-N383-*Ps*POx (from 29 to 0.23 M^−1^ s^−1^) as well as for the line N35-N202-*Sc*POx (from 29 to 0.17 M^−1^ s^−1^). This decrease can mainly be attributed to a gradual shift toward very unfavorable *K*_*m*_ values (0.27, 2,100, and 260 mM for N35, *Sc*POx, and *Ps*POx, respectively). In contrast, the catalytic efficiency for homoorientin increased from N35 along these two lines, at least to some extent. This increase is more pronounced for the ancestors along the various nodes, not so much for the extant enzyme *Ps*POx (5,500, 100,000, and 23,000 M^−1^ s^−1^ for N35, N383, and *Ps*POx, respectively) and not at all for *Sc*POx (5,500, 10,000, and 410 M^−1^ s^−1^ for N35, N202, and *Sc*POx, respectively). This shift in the substrate preference along the evolutionary line can be seen more clearly when regarding the selectivity ratio (18), i.e., the ratio of the catalytic efficiency for the two substrates homoorientin (homo) and glucose (Glc), (*k_cat,homo_*/*K*_*m,homo*_) · (*k_cat,Glc_*/*K*_*m,Glc*_)^−1^. This ratio increases from 147 for N35 to 2,080 for *Sc*POx and 62,100 for *Ps*POx. An overview of the changes in the ratio of kinetic constants for the two substrates homoorientin and glucose is given in Fig. 3, which illustrates that during evolution the substrate preference shifted significantly toward the *C*-glycoside. A comparable shift can be seen for the reactivity with the *O*-glycoside fraxin (frax). Here, activity was only found with the oldest ancestor N35 and in the evolutionary clade IV line N284-N327-N383-*Ps*POx, while none of the other enzymes tested oxidized this glycoside. The selectivity ratio (*k_cat,frax_*/*K*_*m,frax*_) · (*k_cat,Glc_*/*K*_*m,Glc*_)^−1^ increased stepwise from 26.9 for N35 to a maximum value of 2,330 for N383, and then decreased for the extant member of this line, *Ps*POx, to 270. Again, these data show a gradual shift in substrate selectivity from glucose to a glycoside. Activity with puerarin was only found in N35 and the clade III line, and here the selectivity ratio of puerarin to D-glucose shifted from 31 for N35 to 3,300 for *Sc*POx.

**TABLE 1:** Steady-state kinetic parameters of pyranose oxidase enzymes studied and compared (*Tm*POx (1), N35, N67, *Ka*POx (5), N167, N202, *Sc*POx (6), N284, N327, N383, *Ps*POx (4, 10), and CarA (10))^*c*^^,*d*^

	D-glucose			Homoorientin			Fraxin			Puerarin			Aspalathin			Carminic acid			Mangiferin		
	*k*_cat_/s^−1^	*K*_*m*_/mM	*k*_cat_/*K*_*m*_/M^−1^ s^−1^	*k*_cat_/s^−1^	K_m_/mM	*k*_cat_/*K*_*m*_/M^−1^ s^−1^	*k*_cat_/s^−1^	*K*_*m*_/mM	*k*_cat_/*K*_*m*_/M^−1^ s^−1^	*k*_*cat*_ */s* ^*−1*^	*K*_*m*_/mM	*k*_cat_/*K*_*m*_/M^−1^ s^−1^	*k*_cat_/s^−1^	*K*_*m*_/mM	*k*_cat_/*K*_*m*_/M^−1^ s^−1^	*k*_cat_/s^−1^	*K*_*m*_/mM	*k*_cat_/K_m_/M^−1^ s^−1^	*k*_cat_/ s^−1^	K_m_/ mM	*k*_cat_/K_m_/ M^−1^ s^−1^
***Tm*POx** (1)	920*	0.66 ± 0.05*	1.4 × 10^6^*	-	-	-	-	-	-	-	-	-	-	-	-	-	-	-	-	-	-
***Ka*POx** (5)	15 ± 0*	1.5 ± 0.1*	10. × 10^3^ *	-	-	-	-	-	-	-	-	-	-	-	-	-	-	-	-	-	-
**N35**	0.0080	0.27 ± 0.10	29	0.034	0.0062 ± 0.0003	5.5 × 10^3^	0.037	0.044 ± 0.011	840	0.0058	0.0064 ± 0.0001	910	-	-	-	-	-	-	-	-	-
	0.0027*	41 ± 7*	0.066*	-	-	-	-	-	-	-	-	-	-	-	-	-	-	-	-	-	-
**N67**	0.020*	31 ± 8*	0.65*	0.011*	0.12 ± 0.05*	91*	-	-	-	-	-	-	0.027*	0.052 ± 0.007*	520*	-	-	-	-	-	-
**N202**	0.43	440 ± 120	0.98	0.12	0.012 ± 0.004	10 × 10^3^	-	-	-	0.019	0.0058 ± 0.0019	3.3 × 10^3^	-	-	-	-	-	-	-	-	-
***Sc*POx** (6)	0.36	2,100 ± 300	0.17	0.065	0.16 ± 0.04	410	-	-	-	0.073	0.12 ± 0.02	610	-	-	-	-	-	-	-	-	-
**N284**	0.19	24 ± 6	7.9	1.5	0.047 ± 0.009	32 × 10^3^	0.057	0.062 ± 0.027	920	-	-	-	-	-	-	-	-	-	-	-	-
**N327**	0.24	280 ± 80	0.86	3.4	0.065 ± 0.016	52 × 10^3^	0.021	0.14 ± 0.05	150	-	-	-	-	-	-	-	-	-	-	-	-
**N383**	0.21	350 ± 80	0.60	4.9	0.047 ± 0.008	100 × 10^3^	0.18	0.12 ± 0.03	1.5 × 10^3^	-	-	-	-	-	-	-	-	-	-	-	-
***Ps*POx** (4, 10)	0.060	260 ± 90	0.23	1.6^*a*^	0.070± 0.020^*a*^	23 × 10^3*a*^	0.017	0.22 ± 0.02	80	-	-	-	-	-	-	-	-	-	8.1 ± 1.7^*b*^	0.49 ± 0.10^*b*^	(16 ± 2) ×10^3*b*^
**CarA** (7)	-	-	-	-	-	-	-	-	-	-	-	-	-	-	-	4.3 ± 0.1	0.019 ± 0.001	226 × 10^3^	-	-	-

^*a*^ The catalytic constant of the *Ps*POx dehydrogenase activity for homoorientin oxidation using the colorimetric assay was significantly lower than when following oxygen consumption. A *k_cat_* of 31 s^−1^ was obtained when the activity was measured by following oxygen consumption in an oxygraph, resulting in a *k_cat_*/*K*_*m*_ of (3.4 ± 0.3) × 10^5^ M^−1^ s^−1^.

^*b*^ Activity measured by following the oxygen consumption in an oxygraph.

^*c*^ Values were not determined.

^*d*^ The steady-state parameters were measured using the dehydrogenase assay with DCIP as electron acceptor unless labeled with *, which indicates the use of the oxidase assay.

### Comparison of structural predictions

**Fig 4:**
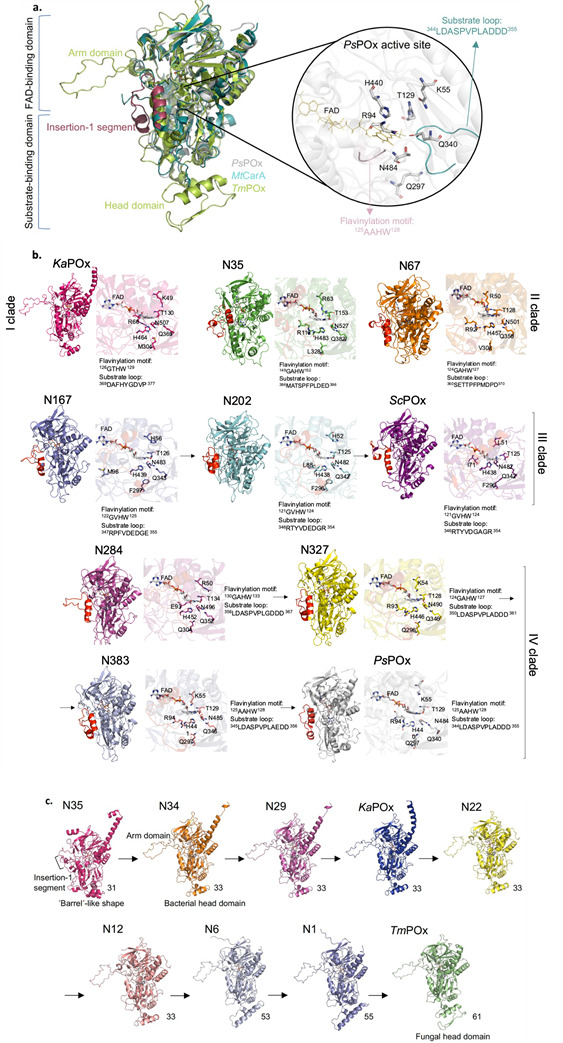
(**a**) Comparison of structures of bacterial POx from *Pseudoarthrobacter siccitolerans* (*Ps*POx) (10) (PDB code 7QF8) (gray), bacterial FAD-dependent C-glycoside 3-oxidase from *Microbacterium trichothecenolyticum* (*Mt*CarA) (7) (PDB code 7DVE) (turquoise), and one subunit of fungal POx from *Trametes multicolor* (*Tm*POx) (19) (PDB code 1TT0) (green). Oligomerization domains from the *Tm*POx structure annotated as head and arm domain are highlighted as is the insertion-1 segment, a structural motif from the *Ps*POx crystal structure. The magnified part of the *Ps*POx active site shows residues important for forming the enzyme-substrate complex and catalysis. The FAD molecule is colored in yellow, the substrate loop in blue, and the flavinylation motif in pink. Catalytically important residues are colored according to the type of atom. (**b**) Comparison of structural predictions of the ancestors N35, N67, N167, N202, N284, N327, and N383 as well as present-day POx from *Kitasatospora aureofaciens* (*Ka*POx) (5) and *Streptomyces canus* (*Sc*POx) (6) with *P. siccitolerans* (*Ps*POx). The FAD molecule (from the structure of *Ps*POx) is highlighted in all structures. The insertion-1 segment is colored red in proteins where it is present, and the segment was identified based on the sequence alignment in Fig. S7. Sequences predicted for the flavinylation motif and the substrate loop are identified next to the models. As these regions are very flexible, only their primary sequence is shown. Catalytically important residues and FAD (from the *Ps*POx crystal structure) are colored according to the type of atom. Clade labels are also included next to the structural predictions. **(c**) Comparison of the structural predictions of monomeric N35, N34, N29, *Ka*POx, N22, N12, N6 and N1 with the crystal structures of *Ps*POx (PDB code 7QF8) and the *Tm*POx monomer (PDB code 1TT0). The arm and head domains as well as the insertion-1 segment are indicated. The number of amino acids forming the head domain is next to the structural predictions and is based on Fig. S8. FAD (from the structure of *Tm*POx) is colored according to the type of atom.

Only recently, the crystal structure of *Ps*POx was reported at 2.01 Å resolution, as were the structures of *Ps*POx in complex with glucose and mangiferin (10). These structures revealed features of the enzyme that are important for substrate binding and reactivity, namely the mobile substrate loop projecting into the active site cavity and the insertion-1 segment interacting with this substrate loop. We took the *Ps*POx structure as template for structural comparison and target-based structural modeling for the various ancestors studied here. The *Ps*POx structure was used for structural comparison with extant isoforms of known structures, *Tm*POx (19) and *Mt*CarA (7) (Fig. 4a).

*Ps*POx, *Mt*CarA, and *Tm*POx display a highly conserved overall fold, with differences mainly found in the head domain, the oligomerization loop (the arm domain), both of which are important for tetramerization of *Tm*POx, or the insertion-1 segment, which occupies the same region as the oligomerization loop but controls the entrance of substrates into the active site of *Ps*POx (Fig. 4a) (10). Even though the sequence identities of some ancestors and the two extant bacterial enzymes were low when compared to the sequence of *Ps*POx (Table S4), the structural predictions of the ancestral enzymes, *Sc*POx and *Ka*POx showed a well-conserved overall fold, an FAD- and a substrate-binding domain, with the characteristic Rossmann fold-like structure in the FAD-binding domain and a combination of α-helices and β-sheets in the substrate-binding domain (Fig. 4b). Despite this well-conserved overall fold, local similarity data prediction showed that some parts of models of the ancestral enzymes, mostly flexible loops, had only low confidence (Fig. S6). As these loop regions, such as the flavinylation motif, the substrate loop, and the insertion-1 segment, are known to play important roles in substrate specificity and binding, we investigated them in more detail based on sequence alignment (a full-length sequence alignment is shown in Fig. S7). Differences in the number of mutations of target ancestors compared to their extant forms and number of consensus mutations are summarised in Table S5.

In *Ps*POx, the access of substrate to the isoalloxazine (within the active site) is through a solvent-accessible cavity that is lined by the flavinylation motif (^125^AAHW^128^) non-covalently binding the cofactor, the substrate loop (^344^LDASPVPLADDD^355^) and the insertion-1 segment (^60^PDSRSLAQRASEGPGAGAATVNSPGAVKSGERRA^93^) (Fig. 4a) (10). Both the sequences of the flavinylation motif and the substrate loop show distinct differences between the different POx clades (Fig. 4b). These (consensus) sequences for the flavinylation motif and the substrate loop are GTWH and DAFHYGDVP in clade I, GAWH and SETTPFPMDP in clade II, GVHW and R(P/T) (F/Y)VDEDG(E/R) in clade III, and (G/A)AHW and LDASPVPL(A/G) (D/E)DD in clade IV, respectively. The Thr residue immediately following the flavinylation motif, which was shown to form a H-bond to N5 of the isoalloxazine ring in *Mt*CarA (7), is conserved in all sequences (Fig. S7). The sequence of the insertion-1 segment is not well conserved among ancestors and extant POx enzymes; however, enzymes belonging to the same clade show a similar fold of this insertion-1 segment in the models. The structural prediction of *Ka*POx, which is dimeric, does not indicate the presence of a comparable insertion-1 domain but oligomeric domains (arm and head domains), comparable to fungal *Tm*POx. Models of N35 and N67 do not indicate oligomerization domains but rather showed a partial or full insertion-1 segment, even though they presented higher oligomerization states in solution (dimer, or a mixture of trimer pentamer) (Table S2).

Taborda et al. (10) provided structural evidence that substrate binding in the active site of *Ps*POx and catalysis are orchestrated by residues K55, R94, T129, Q297, Q340, H440, and N484 (Fig. 4a). These residues form interactions (hydrogen bonds) with either the D-glucose moiety or the mangiferin aglycone. H440 and N484 play a key role in catalysis and are strictly conserved in the ancestors and extant POx enzymes (Fig. S7). Residue K55 in *Ps*POx was shown to be positioned close to the aglycone part of mangiferin, and residues R94 and Q297 were suggested by both structural analysis and MD simulations to anchor the bulky glycoside substrate near the active site (10). K55 is conserved in the ancestors of clade IV (or replaced by the conservative substitution Lys –> Arg in N284 and N35), while *Sc*POx and clade III ancestors have a His at this position. Similarly, R94 is conserved in clade IV and N35 but replaced by a Glu or Thr residue in clade III, and Q297 is mainly conserved in clade IV but replaced by His in N284 and all the other bacterial enzymes. These differences in residues that directly interact with the electron donor substrate could be responsible for the variability in the reactivity with different glycoside substrates that can be observed between different clades. Figure 4b gives an overview of residues in the vicinity of the active site, taking up positions identical to the one described for *Ps*POx playing an important role in substrate binding and catalysis.

Finally, we were interested in how the split between the two distinct classes of POx—monomeric, bacterial enzymes active mainly on glycosides and oligomeric, bacterial (or fungal) enzymes acting on monosaccharides—took place during evolution. To this end, we modeled the structures of ancestors at nodes linking the oldest ancestor N35 to fungal POx, namely N1, N6, N12, N22, N29, and N34 including present-day *Ka*POx (Fig. 1). The major differences identified in the structural predictions of bacterial and fungal POx are concerning the head and arm domain, the substrate loop, and the insertion-1 segment, and therefore we focused on these regions in the structural comparison (Fig. 4c; Fig. S8). When comparing the structural predictions of N1, N6, N12, N22, *Ka*POx, N29, and N34 to those of N35 (the last common ancestor of all bacterial members of this sequence space), *Ps*POx and *Tm*POx, we observed that all of these possess pronounced arm and head domains, which are important for oligomerization as seen in fungal POx and *Ka*POx, and a substrate loop that differs from that of other bacterial POx or CGOx (Fig. 4c). While the size of the arm domain does not change along the line from N34 to *Tm*POx, the head domain increases in size (from 31 residues in N35 to 55 in N1 and 61 in *Tm*POx), and furthermore, it changes its conformation along the phylogenetic line N34, N29, *Ka*POx, N22, N12, N6, N1, and *Tm*POx. The structural prediction of N35 does not show the pronounced arm and head domains but rather an insertion-1 segment resembling a rotated arm domain, and a “barrel”-shaped bottom, which then seems to have slowly evolved to the head domain of fungal POx. The solvent-accessible surface area of monomers and dimers of N35, N34, N29, *Ka*POx, N22, N12, N6, and N1 displayed a general increasing tendency along the phylogenetic line *Ps*POx, N35, N34, N29, *Ka*POx, N22, N12, N6, N1, and *Tm*POx when comparing it to the same property of crystal structures of *Ps*POx and *Tm*POx (data not shown).

## DISCUSSION

First studies on pyranose oxidase were performed on enzymes of exclusively fungal origin, and these were shown to efficiently oxidize D-glucose as well as other monosaccharides typically found in lignocellulose (D-xylose, L-arabinose, and D-galactose) preferentially at the C2 position but sometimes also at C3 or at both positions (20, 21). When the first bacterial POx, an enzyme from *Pseudoarthrobacter siccitolerans* (*Ps*POx) (4), was reported in 2016 it was puzzling to see some of its catalytic properties, above all the very unfavorable kinetic constants for D-glucose with a Michaelis constant of 460 mM and low catalytic efficiency of 0.45 M^−1^ s^−1^ (10) as glucose had been considered the natural substrate of pyranose oxidases. This, however, could be explained after Kumano et al. isolated a *C*-glycoside-catabolising microorganism, *Microbacterium* sp., from soil that can break the C-C bond in carminic acid by a two-step mechanism (7). After initial oxidation of the sugar moiety, *C*-glycoside deglycosidases cleave the C-C bond between sugar and aglycone by acid/base catalysis (8). The enzyme initiating this deglycosylation reaction in *Microbacterium* sp. was shown to oxidize the glucose moiety of carminic acid at the C3 position (and to some extent also at C2) and termed “*C*-glycoside oxidase” or CarA (7). Subsequently, we showed that CarA belongs to the sequence space of POx, a member of the well-studied GMC superfamily (6), and *Ps*POx was proven to react favorably with another *C*-glycoside, mangiferin (*K*_*m*_ of 0.49 mM, *k_cat_*/*K*_*m*_ of 19,200 M^−1^ s^−1^). Since both fungal, monosaccharide-oxidizing POx (as well as the monosaccharide-oxidizing enzyme from the actinomycete *K. aureofaciens* (5)) and bacterial, glycoside-oxidizing pyranose oxidases belong to the same sequence space they must have evolved from the same ancestral enzyme. It was hence the objective of this study to investigate the phylogeny of actinobacterial POx and the historical trajectory of substrate preference throughout different clades in detail.

The sequence space of actinobacterial POx is clearly divided into four clades as confirmed by high bootstrap values (Fig. 1). An initial screening for substrate reactivity (Fig. 2a and b) gave a clear distinction between fungal *Tm*POx as well as clade I *Ka*POx and the other present-day bacterial enzymes, *Ps*POx and *ScP*Ox, as well as ancestors positioned in clades II–IV. The former only reacted with monosaccharides [specific activities with D-glucose of 12 U/mg for *Tm*POx and 7.6 U/mg for *Ka*POx (1, 5)] and showed no reactivity with any of the glycosides tested. At the same time, the latter only had very low activities with monosaccharides (0.001–0.35 U/mg for D-glucose) and most enzymes oxidized various glycosides with higher efficiency. This initial screening also showed significant differences among clades II–IV with respect to the glycosides accepted. The *C*-glycosides homoorientin and isovitexin were oxidized by all ancestors and extant members of these clades, the *C*-glycoside puerarin mainly by the extant member *Sc*POx and ancestors belonging to clade III (as well as by the oldest common ancestor N35 and the oldest member of the ancestral line of clade IV, N284), and the *O*-glycoside fraxin was only oxidized by extant member *Ps*POx and the ancestors of clade IV (as well as by the oldest common ancestor N35). The oldest common ancestor N35 showed the most diverse substrate reactivity, which corroborates what has been found for other ancestors as well, namely that they can be multifunctional and promiscuous (22), before they eventually evolve into more specialized and efficient enzymes. N35 was also the generalist among the ancestors with respect to activity with glycosides and monosaccharides, judging from the selectivity ratio, i.e., the ratio of the catalytic efficiency for the two substrates homoorientin (homo) and glucose (Glc), (*k_cat,homo_*/*K*_*m,homo*_) (*k_cat,Glc_*/*K*_*m,Glc*_)^−1^. This selectivity ratio increased in both the ancestral lines and extant members of POx clades III and IV, both because of an increase in the Michaelis constant for D-glucose and an increase in the catalytic constant of the glycoside, which is especially notable in clade IV (*k_cat,homo_* values of 0.03, 4.89, and 1.6 s^−1^ for N35, N383 and *Ps*POx, respectively). POx members of clades III and IV show very low *K*_*m*_ values for homoorientin in the micromolar range, which could reflect the low concentrations of glycosides encountered by bacterial organisms in their natural habitats. N67, the only ancestor of clade II studied in this work, shows a reduced selectivity ratio for homoorientin and D-glucose compared to N35, and hence shows comparable but rather low catalytic efficiencies for these two reference substrates. Unfortunately, no present-day POx member of clade II has been studied so we cannot say if these properties are also reflected in extant enzymes of this clade. The extant member of POx clade I, *Ka*POx, completely lost reactivity with any of the glycosides tested in this study and became specialized for the oxidation of monosaccharides as did the fungal enzyme *Tm*POx.

We recently speculated that the catalytic mechanisms and binding specificities within the POx family of enzymes are modulated through different homooligomerization states (10); i.e., the loss of reactivity with the bulkier glycosides is caused by oligomerization*—Ka*POx is a dimer and *Tm*POx is a homotetramer. The structures of *Ps*POx and *Mt*CarA show an open active site with access only controlled by the substrate loop. In contrast, the crystal structure of *Tm*POx indicates a very restricted access to the FAD through an internal void and a narrow substrate channel (23, 24). Here, the oligomerization of clade I POx seems to be initiated by a gradual extension of the head domain, which is important for subunit interaction in *Tm*POx (19), as well as by the evolution of the insertion-1 segment into an arm domain, supporting well the hypothesis that different functional oligomeric states contribute to enzyme specificity. Changes in both domains are supported by structural predictions of the evolutionary line of clade I POx (Fig. 4c; Fig. S8).

In this study, we only focused on sequences of actinobacterial (Actinomycetota) origin since all hitherto characterized bacterial enzymes of the POx family are obtained from species of this phyla. Genes coding for pyranose oxidases were also identified in other phyla of bacteria, such as the Pseudomonadota (classes Alpha- and Gammaproteobacteria) (6). These enzymes have not been studied biochemically in detail, yet various *Rhizobium*, *Agrobacterium*, and *Stenotrophomonas* species (phylum Pseudomonadota) and *Deinococcus aerius* (phylum Deinococcota) were shown to contain genes that are phylogenetically related to bacterial *pox* genes. Furthermore, the corresponding gene products were able to oxidize either various glycosides (25) or glucose (6). FAD-dependent oxidoreductases from *Rhizobium*, *Agrobacterium*, and *Stenotrophomonas* species oxidized the sugar moieties of a range of different ginsenoides, resulting in their deglycosylation. Because of this wide unexplored number of POx sequences, we can expect much wider reactivities of bacterial POx/CGOx with glycosides than currently known.

### Conclusion

Actinobacteria (Actinomycetota) are found in soil or water where they contribute to the degradation of organic matter including plant material, which may contain lignocellulosic material or various glycosides. We conclude from our results that an ancestor of pyranose oxidase, a generalist oxidizing both monosaccharides and various glycosides, evolved into various clades of specialized enzymes that oxidize primarily monosaccharides or glycosides. Fungi sharing the habitat with Actinobacteria may have acquired *pox* genes from organisms forming monosaccharide-oxidizing POx by horizontal transfer.

## MATERIALS AND METHODS

### Generation of enzyme clusters and ancestral sequence calculation

PSI-BLAST (26) searches in the NCBI database were conducted in April and May 2020. The following sequences (accession numbers and annotation) from published data sets (5) were used as seed sequences for the initial search: A0A2G7ETB5 (choline dehydrogenase-like flavoprotein from *Streptomyces* sp.), A0A101RTT1 (choline dehydrogenase from *S. canus*), A0A0S9PHX4 (choline dehydrogenase from *Agreia* sp.), A0A1M5HNJ5 (choline dehydrogenase from *Geodermatophilus nigrescens*), F3P4S3 (conserved domain protein from *Actinomyces* sp.), A0A0Q8AVH9 (choline dehydrogenase from *Microbacterium* sp.), A0A260UCT4 (choline dehydrogenase from *Rhodococcus fascians*), A0A164DUC4 (6′′′-hydroxyparomomycin C oxidase from *Agromyces* sp.), A0A1H5VE67 (pyranose oxidase from *Saccharopolyspora jiangxiensis*), and K4QWW1 (GMC_oxred_C domain-containing protein from *Streptomyces davaonensis*). The search was restricted to Actinobacteria (taxon identifier 201174). Sequences showing identities of 35–99% as well as a threshold value of 0.005 and an *E*-value from 0 to 1e−50 were used as templates for the next iteration step (PSI-BLAST Iteration 2). Sequences from the second iteration with identities of 35–99%, a threshold value of 0.005, a query coverage 60–100% and an *E*-value from 0 to 1e−50 were selected as data set for the ancestral sequence reconstruction. The algorithm Usearch (v.11.0.667) was performed (27), allowing sequences with identity cut-offs higher than 97% being discarded. Sequences shorter than 420 amino acids were also deleted from the data set manually. The tool SeqScrub (28) (http://seqscrub.gabefoley.com/, accessed in September 2021) was used for uniformly renaming all sequences as well as discarding sequences with illegal amino acid characters. After inferring a multiple sequence alignment with MAFFT (v.7.0.26) using the method G-INS-1 (29), sequences not containing GMC structural motifs (2) were discarded from the data set. Trimming of the alignment was performed using Gblocks (v.0.91.1) (30). A substitution model was assessed using ModelTest (v.3.7) (31), and when using the Bayesian and Akaike information criteria the best model predicted was Le-Gascuel (32). The phylogenetic tree was calculated using PhyML (v.3.3_1) (33) using the Le-Gascuel substitution model. Both Gblocks and PhyML were accessed in September 2021 through the web interface NG Phylogeny (34) (https://ngphylogeny.fr/, accessed in September 2021). The tree was rooted on the midpoint and edited using the software FigTree (v.1.4.4) (The University of Edinburgh, UK). Bootstrapping of the tree was performed using RAxML (v.8) (35), where the tree converged after 570 iterations.

The tool Graphical Representation of Ancestral Sequence Predictions (GRASP) (36) (http://grasp.scmb.uq.edu.au/, accessed in September 2021) was used for inferring ancestral sequences using marginal reconstruction and Le-Gascuel as the evolutionary model, with the PhyML-constructed tree as input. Based on their position in the phylogenetic tree, seven ancestors were chosen for further analysis. The sequences were downloaded from GRASP, together with a list of posterior probabilities for each position in the primary sequence. The mean probability for each ancestor was calculated based on aligning the primary sequence to a matching probability calculated by GRASP. The probability of each position was depicted using the software SigmaPlot (v.14.0) (Systat Software, Düsseldorf, Germany). Amino acid sequences of all target ancestors are in Table S6.

### Synthesis and cloning of ancestral sequences

Structural predictions for the ancestors were predicted using RoseTTAFold (https://robetta.bakerlab.org/submit.php, accessed in January 2022) (37). The most probable model, annotated as “Model 1,” was used for determining the C-terminal flanking region. Ancestral structural predictions were explored in the software PyMOL Molecular Graphics System (v.2.5.2, educational license) (Schrödinger, New York, NY, USA). C-terminal flanking sequences of each ancestor were determined by aligning structures to the predicted structural prediction of *Sc*POx (6). Coding sequences for each ancestor (excluding the C-terminal flanking sequence), followed by the TEV cleavage site (ENLYFQS) were cloned into the pET21+ vector between the restriction sites *Bam*HI and *Xho*I. Genes and the TEV cleavage site were codon-optimized for *E. coli*. The ribosome binding sequence (nucleotide sequence TTAAGAAGGAGATATACC) was added after the *Bam*HI restriction site in the pET21+ vector backbone. All constructs were ordered from Twist Biosciences (South San Francisco, CA, USA), and contained an ampicillin-resistance marker cassette and a C-terminal His_6_ tag.

### Gene expression, initial screening for the optimal induction and expression strain, and purification of recombinant proteins

Calcium-competent *E. coli* BL21(DE3) and T7 express strains (already transformed with the vector pGro7 overexpressing groES-groEL to increase soluble expression; New England Biolabs, Ipswich, MA, USA) were transformed with the constructs using the heat-shock transformation method. Cells were grown in 250 or 500 mL LB medium with ampicillin (100 µg/mL) inoculated with overnight cultures diluted 1:90. For the expression of the recombinant genes, bacterial cultures were grown at 37°C with agitation (140 rpm) until OD_600_ reached 0.6–1. After that, induction of gene expression was started by adding 10 mM lactose or 1 mM IPTG (Thermo Scientific, Waltham, MA, USA) and the temperature was decreased to 18°C or 30°C, respectively. Expression continued for ~20 or 3 h, respectively. Cell pellets were collected by centrifugation (20 min, 5,000 rpm, 8°C, centrifuge Avanti J-26 XP, rotor JA-10; Beckman Coulter, Brea, CA, USA). Properties of the recombinantly expressed proteins were determined by ProtParam (38) (https://web.expasy.org/protparam/, accessed in February 2022). All chemicals were from Carl Roth (Karlsruhe, Germany) unless indicated otherwise.

Initial screening was done on a 5-mL scale. The overproduction of the seven ancestral proteins was induced with lactose and IPTG in two different strains, BL21(DE3) and T7(pGro7). Cell pellets were disrupted using sonication with an ultrasonic homogenizer (Sonoplus; Bandelin, Berlin, Germany) at 120 V and 30% cycle for 5 min, repeated three times with 5 min breaks. The soluble fraction was separated from cell debris by centrifugation (1 h, 20,000 rpm, 4°C, centrifuge Avanti J-26 XP, rotor JA-25.50). Initial activities were tested with D-glucose (final concentration 500 mM), mangiferin or puerarin (each at a final concentration at 0.1 mM) using the DCIP (dichlorophenol indophenol; Sigma-Aldrich, St. Louis, MO, USA) and AmplexRed (10-acetyl-3,7-dihydroxyphenoxazine; Chemodex, St. Gallen, Switzerland) assays. The best-performing expression strain and induction strategy were selected based on the highest activity with 500 mM D-glucose in the DCIP assay. Assays for initial screening were performed as described below.

Batch cultivations with 5 L of medium were used to produce protein for subsequent purification by affinity chromatography (ÄKTA Go chromatography system; Cytiva, Marlborough, MA, USA). Cell disruption and separation of soluble fractions from cell debris were done as previously described (6). His-Trap columns (5 mL volume; Cytiva) were equilibrated with purification buffer (150 mM NaCl, 5% glycerol, and 50 mM Tris-HCl, pH 7.5) containing 30 mM imidazole. After the sample was loaded onto the column and washing of unbound proteins; the proteins of interest were eluted with a linear gradient (0–100%, 10 min) of purification buffer containing 500 mM imidazole. The selected fractions were pooled, desalted, and concentrated using ultraconcentrators (MWCO 30 kDa; Merck Millipore, Billerica, MA, USA). All other enzymes used in this study, including the extant bacterial enzymes *Ps*POx (Uniprot accession number A0A024H8G7), *Sc*POx (A0A117Q443), and *Ka*POx (A0A1E7NAU4) as well as the fungal enzyme *Tm*POx (Q7ZA32), were purified as previously reported (4–6, 39). SDS-PAGE was performed as described (6) to determine the purity of fractions and concentrated proteins.

### Determination of protein concentration (FAD loading) and oligomeric state; thermostability measurements

Protein concentrations were determined using a diode array spectrophotometer (Agilent Technologies, Santa Clara, CA, USA) and measuring absorbance at 280 nm. Reconstitution of FAD was performed by incubating the enzyme overnight at 4°C and removing the residual, unbound cofactor using ultraconcentrators (MWCO 30 kDa, Merck Millipore). The ratio of FAD-loaded and unloaded protein was calculated by comparing protein concentrations calculated using the absorbance at 450 nm with the extinction coefficient of FAD (11,300 M^−1^ cm^−1^), and total protein concentration calculated using the absorbance at 280 nm.

For determination of the oligomeric state of proteins, the gel-filtration column Superose 12 10/300 (Cytiva) was operated with 50 mM Tris-HCl buffer, pH 7.5 150 mM NaCl at room temperature. To determine the void volume, blue dextran (*M_r_* = 2000 Da) was injected. The standard curve was calculated by running the standard protein mix Gel-filtration standard (Bio-Rad, Hercules, CA, USA) and the molecular mass of proteins was calculated using the standard curve.

The thermal transition temperature, *T_m_*, of proteins was determined using the ThermoFAD assay (21, 40). Protein samples were diluted to 1 mg/mL in 25 µL buffer. Denaturation was performed 25–80°C with increments of 0.5° every minute. Measurements were performed in triplicates using a real-time PCR cycler (iCycler; Bio-Rad) by following the increase in fluorescence signal of the FAD cofactor upon denaturation. The fluorescent signal was detected using the SYBR-green filters (mMyiQ detection system; Bio-Rad).

### Screening for activity, steady-state parameters determination

The monosaccharides D-glucose and D-xylose, the *C*-glycosides aspalathin (PhytoLab, Vestenbergsgreuth, Germany), carminic acid (Glentham Life Sciences, Corsham, UK), homoorientin (isoorientin) (abcr, Karlsruhe, Germany), isovitexin (TargetMol, Boston, MA, USA), mangiferin (Sigma-Aldrich), puerarin (abcr), the *O*-glycosides fraxin (BLDpharm, Puding, China), naringin, salicin, rutin (all from abcr), and one *S*-glycoside, sinigrin (Sigma-Aldrich), were used as possible substrates in the initial activity screening. While the monosaccharides were dissolved in water, a stock solution of the glycosides was prepared in pure DMSO. The DCIP (measuring the dehydrogenase activity) and AmplexRed-coupled (for oxidase activity) assays were performed as previously described (6), however in 200 µL reaction volume and 96-well plates. Activity for *Ka*POx and *Tm*POx was measured using an ABTS (2,2′-azino-bis(3-ethylbenzothiazoline-6-sulfonic acid; Sigma-Aldrich)-coupled oxidase assay according to a published protocol (5). The activities obtained were corrected for the fraction of enzyme containing the cofactor FAD. Heatmaps showing the results from screening for activity were plotted using the Python data visualization library, Seaborn (41).

Apparent steady-state kinetic parameters were determined by varying the concentration of electron donor (monosaccharide or glycoside) while keeping the concentration of electron acceptor constant (DCIP or O_2_, in excess). The catalytic parameters of *Ps*POx for glycosides were also determined by monitoring oxygen consumption using the Oxygraph system (Hansatech Instruments, Pentney, UK), as described in reference (10). Concentrations of substrates used to determine apparent steady-state kinetic parameters were as follows: D-glucose 0–800 mM (except for *Ps*POx 0–1250 mM), aspalathin 0–0.6 mM, fraxin 0–0.6 mM (except for *Ps*POx 0–0.5 mM), homoorientin 0–0.6 mM (except for *Ps*POx 0–0.3 mM), mangiferin 0–1.8 mM, and puerarin 0–0.5 mM. Measurements were performed at 30°C and at pH 7.5 (50 mM Tris-HCl buffer) on the plate readers BioTek (Agilent Technologies) or EnSpire (PerkinElmer, Waltham, MA, USA). All curves were fitted to the Michaelis-Menten equation using the software SigmaPlot, Dynamic Fit Wizard.

### Principal competent analysis

PCA was performed using the web tool ClustVis (42) (https://biit.cs.ut.ee/clustvis/, accessed in March 2023). Activity data used to plot the heatmap were used as a matrix for the analysis. During the pre-processing of raw data, raw scaling was performed using Unit Variance Scaling and the PCA method using Single Variance Decomposition with imputation. The PCA scores and PCA loadings were plotted using principal components 1 and 2 from calculated values.

### Structural modeling and structure analysis

Ancestral amino acid sequences were modeled with the *Ps*POx crystal structure (PDB 7QF8) (10) as a template using SWISS-MODEL (accessed in March 2023) (43). Structural predictions for *Sc*POx and *Ka*POx were taken from previously published studies (5, 6), whereas the structures for *Mt*CarA (PDB 7DVE) (7) and *Tm*POx (PDB 1TT0) (19) were downloaded from RCSB PDB (https://www.rcsb.org/, accessed in March 2023). N1, N6, N12, N22, *Ka*POx, N29, and N34 monomeric structural predictions were obtained using SWISS-MODEL, while their dimeric structural predictions were obtained by aligning monomers to the *Tm*POx dimeric structure. Structural predictions were explored in the software PyMOL. The solvent-accessible surface area was calculated using the built-in PyMOL algorithm. Alignments were prepared in the sequence editor BioEdit (v.7.2) (Tom Hall).

## SUPPLEMENTAL MATERIAL

Tables S1-S6 and Figures S1-S8 aem.01676-23-s0001.docx Tables S1 (Fidelity of the common ancestor reconstruction for ancestors of interest), S2 (Comparison of different *Escherichia coli* expression hosts and type of induction), S3 (Lambda maxima and associated extinction coefficient of UV/Vis absorption spectra), S4 (Average pairwise sequence identity between extant and ancestral primary sequences belonging to the POx and CGOx sequence space), S5 (Difference in number of mutations of target ancestors compared to their extant forms and number of consensus mutations), and S6 (Amino acid sequences of the target ancestors) and Fig. S1 (Phylogeny of the pyranose oxidase [POx] and *C*-glycoside oxidase [CGOx] sequence space), S2 (Barcode graphs displaying posterior probability distribution through the primary sequences), S3 (SDS-PAGE analysis of protein preparations purified by affinity chromatography), S4 (UV/Vis absorption spectra after reconstitution with FAD), S5 (Size exclusion chromatograms of selected target ancestors), S6 (Predicted local similarity statistical data of ancestral structural predictions to the target crystal structure of *Tm*POx and *Ps*POx), S7 (Multiple sequence alignment of the fungal enzymes *Tm*POx (1) and *Phanerochaete chrysosporium* POx, present-day bacterial enzymes *Ka*POx, *Ps*POx, *Sc*POx and bacterial FAD-dependent C-glycoside 3-oxidase from *Microbacterium trichothecenolyticum* (*Mt*CarA), together with ancestral sequences of N35, N67, N167, N202, N284, N327, and N383) and S8 (Multiple sequence alignment of the sequences for fungal *Tm*POx and bacterial *Ka*POx, *Ps*POx, *Sc*POx, and *Mt*CarA, together with ancestral sequences of N1, N6, N12, N22, N29, N34, and N35).

ASM does not own the copyrights to Supplemental Material that may be linked to, or accessed through, an article. The authors have granted ASM a non-exclusive, world-wide license to publish the Supplemental Material files. Please contact the corresponding author directly for reuse.

## References

[1] Leitner C, Volc J, Haltrich D. 2001. Purification and characterization of pyranose oxidase from the white rot fungus Trametes multicolor. Appl Environ Microbiol 67:3636–3644. doi:10.1128/AEM.67.8.3636-3644.2001 11472941 PMC93065

[2] Sützl L, Foley G, Gillam EMJ, Bodén M, Haltrich D. 2019. The GMC superfamily of oxidoreductases revisited: analysis and evolution of fungal GMC oxidoreductases. Biotechnol Biofuels 12:118. doi:10.1186/s13068-019-1457-0 31168323 PMC6509819

[3] Abrera AT, Sützl L, Haltrich D. 2020. Pyranose oxidase: a versatile sugar oxidoreductase for bioelectrochemical applications. Bioelectrochemistry 132:107409. doi:10.1016/j.bioelechem.2019.107409 31821902

[4] Mendes S, Banha C, Madeira J, Santos D, Miranda V, Manzanera M, Ventura MR, van Berkel WJH, Martins LO. 2016. Characterization of a bacterial pyranose 2-oxidase from Arthrobacter siccitolerans. J Molecular Catalysis B: Enzy 133:S34–S43. doi:10.1016/j.molcatb.2016.11.005

[5] Herzog PL, Sützl L, Eisenhut B, Maresch D, Haltrich D, Obinger C, Peterbauer CK. 2019. Versatile oxidase and dehydrogenase activities of bacterial pyranose 2-oxidase facilitate redox cycling with manganese peroxidase in vitro. Appl Environ Microbiol 85:e00390-19. doi:10.1128/AEM.00390-19 31028028 PMC6581175

[6] Kostelac A, Sützl L, Puc J, Furlanetto V, Divne C, Haltrich D. 2022. Biochemical characterization of pyranose oxidase from Streptomyces canus—towards a better understanding of pyranose oxidase homologues in bacteria. Int J Mol Sci 23:13595. doi:10.3390/ijms232113595 36362382 PMC9659204

[7] Kumano T, Hori S, Watanabe S, Terashita Y, Yu HY, Hashimoto Y, Senda T, Senda M, Kobayashi M. 2021. FAD-dependent C-glycoside-metabolizing enzymes in microorganisms: screening, characterization, and crystal structure analysis. Proc Natl Acad Sci U S A 118:e2106580118. doi:10.1073/pnas.2106580118 34583991 PMC8501837

[8] Mori T, Kumano T, He H, Watanabe S, Senda M, Moriya T, Adachi N, Hori S, Terashita Y, Kawasaki M, Hashimoto Y, Awakawa T, Senda T, Abe I, Kobayashi M. 2021. C-glycoside metabolism in the gut and in nature: identification, characterization, structural analyses and distribution of C-C bond-cleaving enzymes. Nat Commun 12:6294. doi:10.1038/s41467-021-26585-1 34728636 PMC8563793

[9] Wei B, Wang YK, Qiu WH, Wang SJ, Wu YH, Xu XW, Wang H. 2020. Discovery and mechanism of intestinal bacteria in enzymatic cleavage of C–C glycosidic bonds. Appl Microbiol Biotechnol 104:1883–1890. doi:10.1007/s00253-019-10333-z 31932892

[10] Taborda A, Frazão T, Rodrigues MV, Fernández-Luengo X, Sancho F, Lucas MF, Frazão C, Melo EP, Ventura MR, Masgrau L, Borges PT, Martins LO. 2023. Mechanistic insights into glycoside 3-oxidases involved in C-glycoside metabolism in soil microorganisms. Nat Commun 14:7289. doi:10.1038/s41467-023-42000-3 37963862 PMC10646112

[11] Merkl R, Sterner R. 2016. Reconstruction of ancestral enzymes. Perspectives in Sci 9:17–23. doi:10.1016/j.pisc.2016.08.002

[12] Gumulya Y, Gillam EMJ. 2017. Exploring the past and the future of protein evolution with ancestral sequence reconstruction: the “retro” approach to protein engineering. Biochem J 474:1–19. doi:10.1042/BCJ20160507 28008088

[13] Thomson RES, Carrera-Pacheco SE, Gillam EMJ. 2022. Engineering functional thermostable proteins using ancestral sequence reconstruction. J Biol Chem 298:102435. doi:10.1016/j.jbc.2022.102435 36041629 PMC9525910

[14] Babkova P, Sebestova E, Brezovsky J, Chaloupkova R, Damborsky J. 2017. Ancestral haloalkane dehalogenases show robustness and unique substrate specificity. Chembiochem 18:1448–1456. doi:10.1002/cbic.201700197 28419658

[15] Gumulya Y, Baek J-M, Wun S-J, Thomson RES, Harris KL, Hunter DJB, Behrendorff JBYH, Kulig J, Zheng S, Wu X, Wu B, Stok JE, De Voss JJ, Schenk G, Jurva U, Andersson S, Isin EM, Bodén M, Guddat L, Gillam EMJ. 2018. Engineering highly functional thermostable proteins using ancestral sequence reconstruction. Nat Catal 1:878–888. doi:10.1038/s41929-018-0159-5

[16] Thomas A, Cutlan R, Finnigan W, van der Giezen M, Harmer N. 2019. Highly thermostable carboxylic acid reductases generated by ancestral sequence reconstruction. Commun Biol 2:429. doi:10.1038/s42003-019-0677-y 31799431 PMC6874671

[17] Bailleul G, Nicoll CR, Mascotti ML, Mattevi A, Fraaije MW. 2021. Ancestral reconstruction of mammalian FMO1 enables structural determination, revealing unique features that explain its catalytic properties. J Biol Chem 296:100221. doi:10.1074/jbc.RA120.016297 33759784 PMC7948450

[18] Morley KL, Kazlauskas RJ. 2005. Improving enzyme properties: when are closer mutations better Trends Biotechnol 23:231–237. doi:10.1016/j.tibtech.2005.03.005 15866000

[19] Hallberg BM, Leitner C, Haltrich D, Divne C. 2004. Crystal structure of the 270 kDa homotetrameric lignin-degrading enzyme pyranose 2-oxidase. J Mol Biol 341:781–796. doi:10.1016/j.jmb.2004.06.033 15288786

[20] Sützl L, Laurent C, Abrera AT, Schütz G, Ludwig R, Haltrich D. 2018. Multiplicity of enzymatic functions in the CAZy AA3 family. Appl Microbiol Biotechnol 102:2477–2492. doi:10.1007/s00253-018-8784-0 29411063 PMC5847212

[21] Wijayanti SD, Sützl L, Duval A, Haltrich D. 2021. Characterization of fungal FAD-dependent AA3_2 glucose oxidoreductases from hitherto unexplored phylogenetic clades. J Fungi 7:873. doi:10.3390/jof7100873 PMC8537048 34682294

[22] Spence MA, Kaczmarski JA, Saunders JW, Jackson CJ. 2021. Ancestral sequence reconstruction for protein engineers. Curr Opin Struct Biol 69:131–141. doi:10.1016/j.sbi.2021.04.001 34023793

[23] Kujawa M, Ebner H, Leitner C, Hallberg BM, Prongjit M, Sucharitakul J, Ludwig R, Rudsander U, Peterbauer C, Chaiyen P, Haltrich D, Divne C. 2006. Structural basis for substrate binding and regioselective oxidation of monosaccharides at C3 by pyranose 2-oxidase. J Biol Chem 281:35104–35115. doi:10.1074/jbc.M604718200 16984920

[24] Spadiut O, Tan TC, Pisanelli I, Haltrich D, Divne C. 2010. Importance of the gating segment in the substrate-recognition loop of pyranose 2-oxidase. FEBS J 277:2892–2909. doi:10.1111/j.1742-4658.2010.07705.x 20528921

[25] Kim EM, Seo JH, Baek K, Kim BG. 2015. Characterization of two-step deglycosylation via oxidation by glycoside oxidoreductase and defining their subfamily. Sci Rep 5:10877. doi:10.1038/srep10877 26057169 PMC4650693

[26] Bhagwat M, Aravind L. 2007. PSI-BLAST tutorial. Methods Mol Biol 395:177–186. doi:10.1007/978-1-59745-514-5_10 17993673 PMC4781153

[27] Edgar RC. 2010. Search and clustering orders of magnitude faster than BLAST. Bioinformatics 26:2460–2461. doi:10.1093/bioinformatics/btq461 20709691

[28] Foley G, Sützl L, D’Cunha SA, Gillam EM, Bodén M. 2019. SeqScrub: a web tool for automatic cleaning and annotation of FASTA file headers for bioinformatic applications. Biotechniques 67:50–54. doi:10.2144/btn-2018-0188 31218882

[29] Katoh K, Rozewicki J, Yamada KD. 2019. MAFFT online service: multiple sequence alignment, interactive sequence choice and visualization. Brief Bioinform 20:1160–1166. doi:10.1093/bib/bbx108 28968734 PMC6781576

[30] Talavera G, Castresana J. 2007. Improvement of phylogenies after removing divergent and ambiguously aligned blocks from protein sequence alignments. Syst Biol 56:564–577. doi:10.1080/10635150701472164 17654362

[31] Posada D, Crandall KA. 1998. MODELTEST: testing the model of DNA substitution. Bioinformatics 14:817–818. doi:10.1093/bioinformatics/14.9.817 9918953

[32] Le SQ, Gascuel O. 2008. An improved general amino acid replacement matrix. Mol Biol Evol 25:1307–1320. doi:10.1093/molbev/msn067 18367465

[33] Guindon S, Dufayard JF, Lefort V, Anisimova M, Hordijk W, Gascuel O. 2010. New algorithms and methods to estimate maximum-likelihood phylogenies: assessing the performance of PhyML 3.0. Syst Biol 59:307–321. doi:10.1093/sysbio/syq010 20525638

[34] Lemoine F, Correia D, Lefort V, Doppelt-Azeroual O, Mareuil F, Cohen-Boulakia S, Gascuel O. 2019. NGPhylogeny.fr: new generation phylogenetic services for non-specialists. Nucleic Acids Res 47:W260–W265. doi:10.1093/nar/gkz303 31028399 PMC6602494

[35] Stamatakis A. 2014. RAxML version 8: a tool for phylogenetic analysis and post-analysis of large phylogenies. Bioinformatics 30:1312–1313. doi:10.1093/bioinformatics/btu033 24451623 PMC3998144

[36] Foley G, Mora A, Ross CM, Bottoms S, Sützl L, Lamprecht ML, Zaugg J, Essebier A, Balderson B, Newell R, Thomson RES, Kobe B, Barnard RT, Guddat L, Schenk G, Carsten J, Gumulya Y, Rost B, Haltrich D, Sieber V, Gillam EMJ, Bodén M. 2022. Engineering Indel and substitution variants of diverse and ancient enzymes using graphical representation of ancestral sequence predictions (GRASP). PLoS Comput Biol 18:e1010633. doi:10.1371/journal.pcbi.1010633 36279274 PMC9632902

[37] Baek M, DiMaio F, Anishchenko I, Dauparas J, Ovchinnikov S, Lee GR, Wang J, Cong Q, Kinch LN, Schaeffer RD, et al.. 2021. Accurate prediction of protein structures and interactions using a three-track neural network. Science 373:871–876. doi:10.1126/science.abj8754 34282049 PMC7612213

[38] Gasteiger E, Hoogland C, Gattiker A, Duvaud S, Wilkins MR, Appel RD, Bairoch A. 2005. Protein identification and analysis tools on the ExPASy server, p 571–607. In Walker John M (ed), (ed), (ed), (ed), The proteomics protocols handbook. Humana Press, Totowa NJ.

[39] Spadiut O, Nguyen TT, Haltrich D. 2010. Thermostable variants of pyranose 2-oxidase showing altered substrate selectivity for glucose and galactose. J Agric Food Chem 58:3465–3471. doi:10.1021/jf9040047 20158200

[40] Forneris F, Orru R, Bonivento D, Chiarelli LR, Mattevi A. 2009. Thermo FAD, a thermofluor‐adapted flavin ad hoc detection system for protein folding and ligand binding. FEBS J 276:2833–2840. doi:10.1111/j.1742-4658.2009.07006.x 19459938

[41] Waskom M. 2021. seaborn: statistical data visualization. J Open Source Softw 6:3021. doi:10.21105/joss.03021

[42] Metsalu T, Vilo J. 2015. ClustVis: a web tool for visualizing clustering of multivariate data using principal component analysis and heatmap. Nucleic Acids Res 43:W566–W570. doi:10.1093/nar/gkv468 25969447 PMC4489295

[43] Waterhouse A, Bertoni M, Bienert S, Studer G, Tauriello G, Gumienny R, Heer FT, de Beer TAP, Rempfer C, Bordoli L, Lepore R, Schwede T. 2018. SWISS-MODEL: homology modelling of protein structures and complexes. Nucleic Acids Res 46:W296–W303. doi:10.1093/nar/gky427 29788355 PMC6030848

