# Supplementary material for: Evolution and separation of actinobacterial pyranose and C-glycoside-3-oxidases
Source: Appl Environ Microbiol. 2024 Jan 5;90(1):e01676-23. doi: 10.1128/aem.01676-23 (PMC10807413; doi:10.1128/aem.01676-23)
Supplement: Tables S1-S6 and Figures S1-S8 — Tables S1 (Fidelity of the common ancestor reconstruction for ancestors of interest), S2 (Comparison of different Escherichia coli expression hosts and type of induction), S3 (Lambda maxima and associated extinction coefficient of UV/Vis absorption spectra), S4 (Average pairwise sequence identity between extant and ancestral primary sequences belonging to the POx and CGOx sequence space), S5 (Difference in number of mutations of target ancestors compared to their extant forms and number of consensus mutations), and S6 (Amino acid sequences of the target ancestors) and Fig. S1 (Phylogeny of the pyranose oxidase [POx] and C-glycoside oxidase [CGOx] sequence space), S2 (Barcode graphs displaying posterior probability distribution through the primary sequences), S3 (SDS-PAGE analysis of protein preparations purified by affinity chromatography), S4 (UV/Vis absorption spectra after reconstitution with FAD), S5 (Size exclusion chromatograms of selected target ancestors), S6 (Predicted local similarity statistical data of ancestral structural predictions to the target crystal structure of TmPOx and PsPOx), S7 (Multiple sequence alignment of the fungal enzymes TmPOx (1) and Phanerochaete chrysosporium POx, present-day bacterial enzymes KaPOx, PsPOx, ScPOx and bacterial FAD-dependent C-glycoside 3-oxidase from Microbacterium trichothecenolyticum (MtCarA), together with ancestral sequences of N35, N67, N167, N202, N284, N327, and N383) and S8 (Multiple sequence alignment of the sequences for fungal TmPOx and bacterial KaPOx, PsPOx, ScPOx, and MtCarA, together with ancestral sequences of N1, N6, N12, N22, N29, N34, and N35). [file aem.01676-23-s0001.docx]

**SUPPLEMENTARY INFORMATION**

Evolution and separation of actinobacterial pyranose and *C*-glycoside-3-oxidases

Anja Kostelac^1,2^, André Taborda^3^, Lígia O. Martins^3^, Dietmar Haltrich^1^

1 Department of Food Science and Technology, BOKU - University of Natural Resources and Life Sciences, Vienna, Austria

2 Doctoral Programme BioToP - Biomolecular Technology of Proteins, BOKU - University of Natural Resources and Life Sciences, Vienna, Austria

3 Instituto de Tecnologia Química e Biológica António Xavier, Universidade NOVA de Lisboa, Oeiras, Portugal


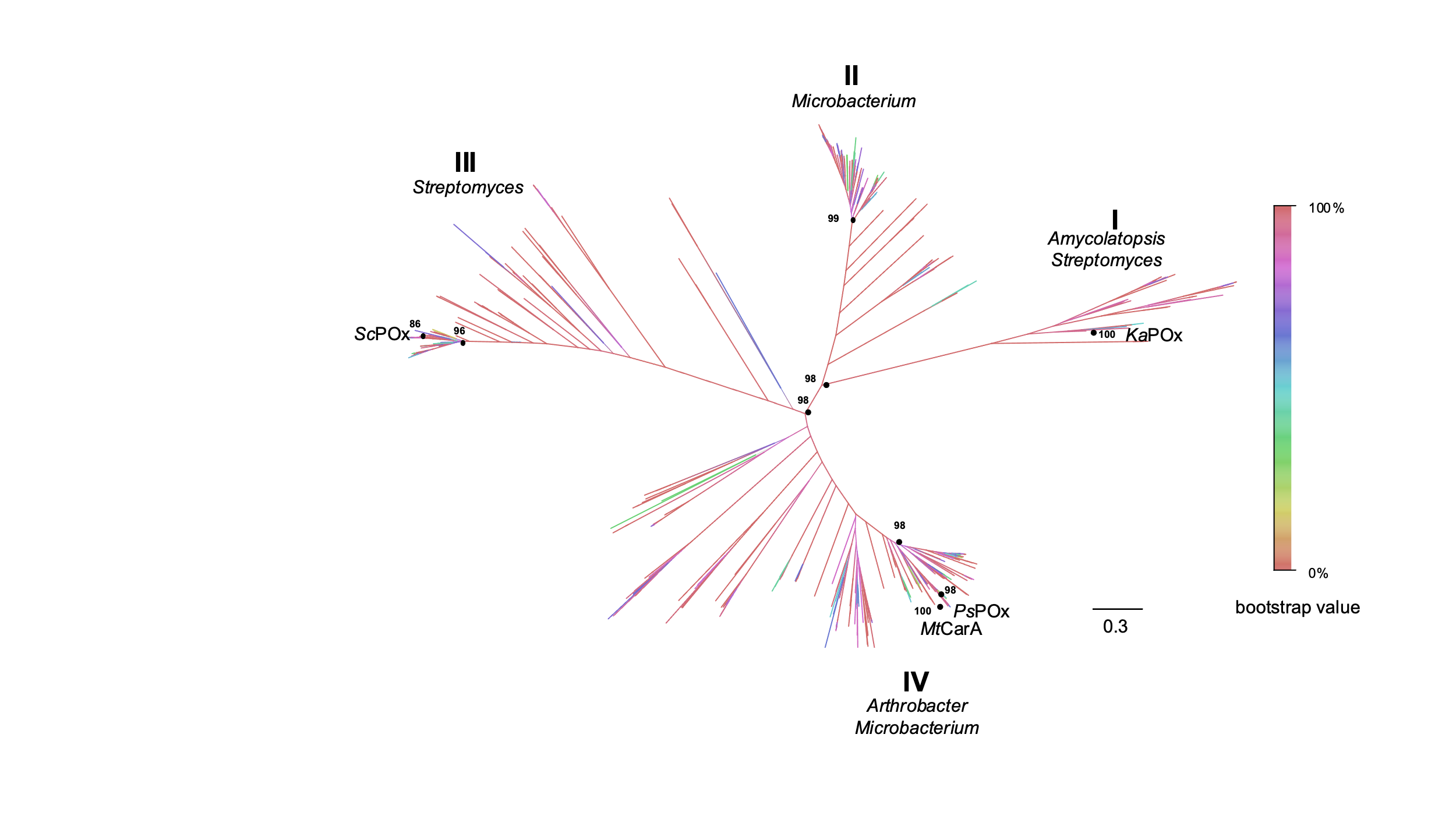


**Figure S1.** Maximum likelihood-constructed phylogeny of the pyranose oxidase (POx) and *C*-glycoside oxidase (CGOx) sequence space by RAxML. No fungal POx sequences (such as the one *Trametes multicolor* POx, *Tm*POx (1)) were included. The bar represents phylogenetic distance as amino acid substitution per site. Bootstrapping values (in %) are shown for selected nodes of interest.

**Table S1.** Fidelity of the common ancestor reconstruction for ancestors of interest. The average posterior probability as well as number of residues unambiguously (states with posterior probability between 80 and 100%) and ambiguously (states with second-highest posterior probability higher than 20%) reconstructed are shown.

| **Ancestor** | **Total** **number of residues** | **Average posterior probability/%** | **Number of residues in ancestral sequence reconstructed**  **(% in the sequence)** | | |
| --- | --- | --- | --- | --- | --- |
|  |  |  | **Unambiguously** | | **Ambiguously** |
|  |  |  | Having posterior probability = 100% | Having posterior probability  ≥ 80% and <100% | Having more than one state with posterior probability  ≥ 20% |
| **N35** | 551 | 83 | 46(8) | 340(62) | 110(20) |
| **N67** | 521 | 98 | 358(69) | 149(29) | 11(2) |
| **N167** | 507 | 93 | 113(22) | 321(63) | 53(10) |
| **N202** | 509 | 99 | 437(86) | 64(12) | 7(1) |
| **N284** | 541 | 90 | 156(29) | 277(51) | 56(10) |
| **N327** | 535 | 95 | 256(48) | 223(41) | 40(7) |
| **N383** | 524 | 97 | 385(73) | 104(20) | 28(5) |

posterior probability/ %

**Figure S2.** Barcode graphs displaying posterior probability distribution through the primary sequences (per site) for N35, N67, N167, N202, N284, N327 and N383.

**Table S2.** Comparison of different *Escherichia coli* expression hosts and type of induction (inducer, duration, temperature) for *Tm*POx (1), N35, N67, *Kitasatospora aureofaciens* POx (*Ka*POx) (2), N167, N202, *Streptomyces canus* POx (*Sc*POx) (3), N284, N327, N383, *Pseudoarthrobacter siccitolerans* (*Ps*POx) (4, 5) and FAD-dependent *C*-glycoside 3-oxidase from *Microbacterium* 5-2b (CarA) (6). The table also includes protein yield after purification per L of medium, thermostability and oligomeric state. The thermal transition temperature, *T_m_*, of proteins was determined with the ThermoFAD assay, and measures the release of FAD. Values in the cells shaded grey were retrieved from literature.

| **Protein** | ***E. coli* expression strain** | **Induction system** | **Yield of active, purified enzyme per 1 L culture/ mg** | **Thermostability (*T_m_*) / °C** | **Oligomeric state** |
| --- | --- | --- | --- | --- | --- |
| ***Tm*POx** (1) | BL21(DE3) | Lactose, 20 h, 25°C | - | 63* | Tetramer |
| **N35** | BL21(DE3) | Lactose, 20 h, 18°C | 0.5 | 52 | Trimer/pentamer |
| **N67** | BL21(DE3) | Lactose, 20 h, 18°C | 1.2 | 51 | Dimer |
| ***Ka*POx** (2) | T7 Express | Lactose, 20 h, 20°C | 13.3 | 53* | Dimer |
| **N167** | T7(pGro7) | IPTG, 3 h, 30°C | 0.2 | 57 | Monomer |
| **N202** | BL21(DE3) | IPTG, 3 h, 30°C | 0.1 | 51 | Monomer |
| ***Sc*POx** (3) | BL21(DE3) | Lactose, 20 h, 18°C | 2.5 | 52 | Monomer |
| **N284** | BL21(DE3) | Lactose, 20 h, 18°C | 0.4 | 46 | Monomer |
| **N327** | BL21(DE3) | Lactose, 20 h, 18°C | 0.3 | 62 | Monomer |
| **N383** | T7(pGro7) | IPTG, 3 h, 30°C | 0.2 | 48 | Monomer |
| ***Ps*POx** (4, 5) | Rosetta pLysS (DE3) | IPTG, 17 h, 25°C | 6 | 38 | Monomer |
| **CarA** (6) | Rosetta2 (DE3) | IPTG, 20 h, 18°C | 0.000435 | - | Monomer |

**Figure S3.** SDS-PAGE analysis of protein preparations purified by affinity chromatography, the total protein amount loaded was 0.9 µg. 1 – protein ladder Precision Plus Protein Standards (BioRad) with indicated protein bands, 2 – N35, 3 – N67, 4 – N167, 5 – N202, 6 – N284, 7 – N327, 8 – N383. Bands representing proteins of interest are indicated with arrows.

**N67**

N67

**N35**


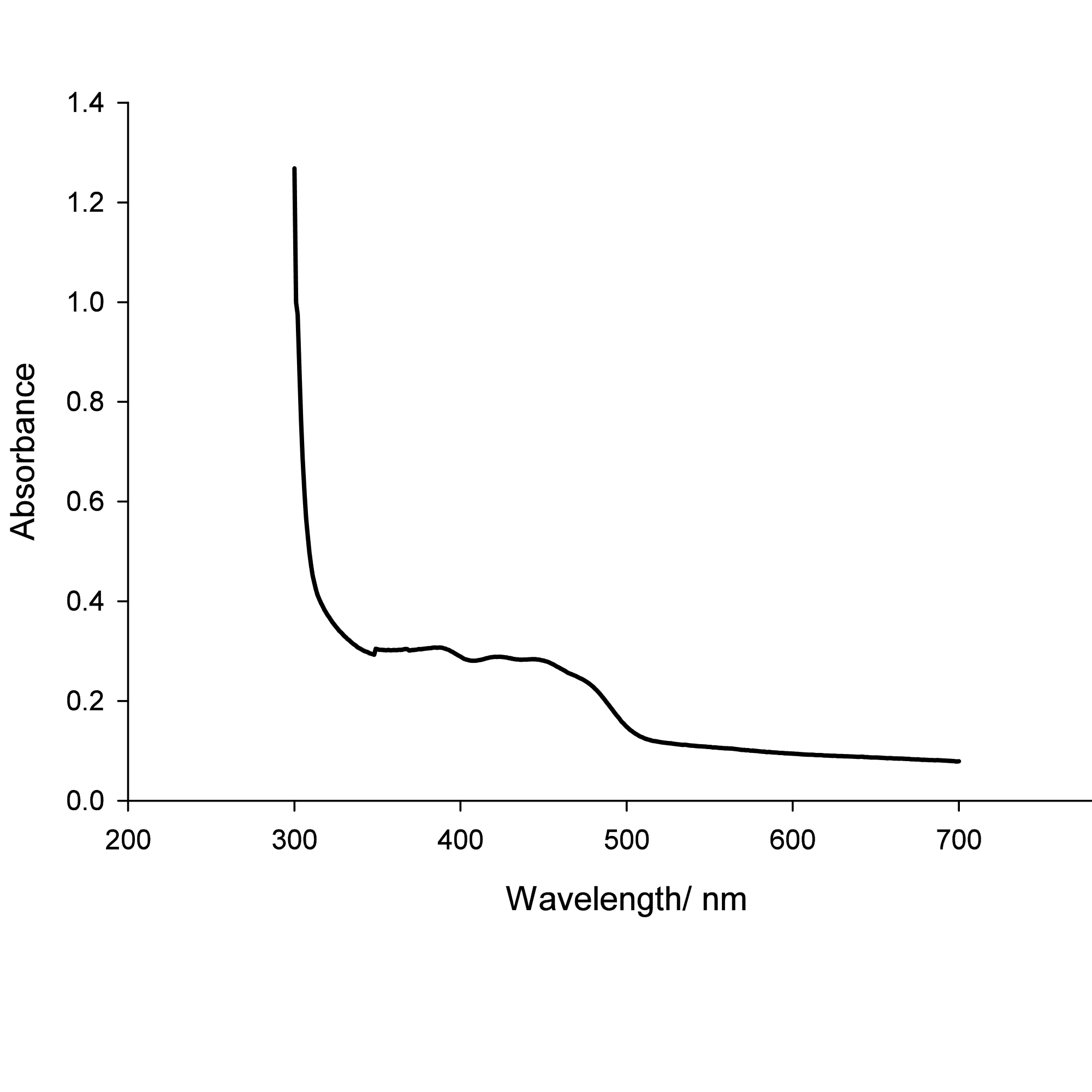

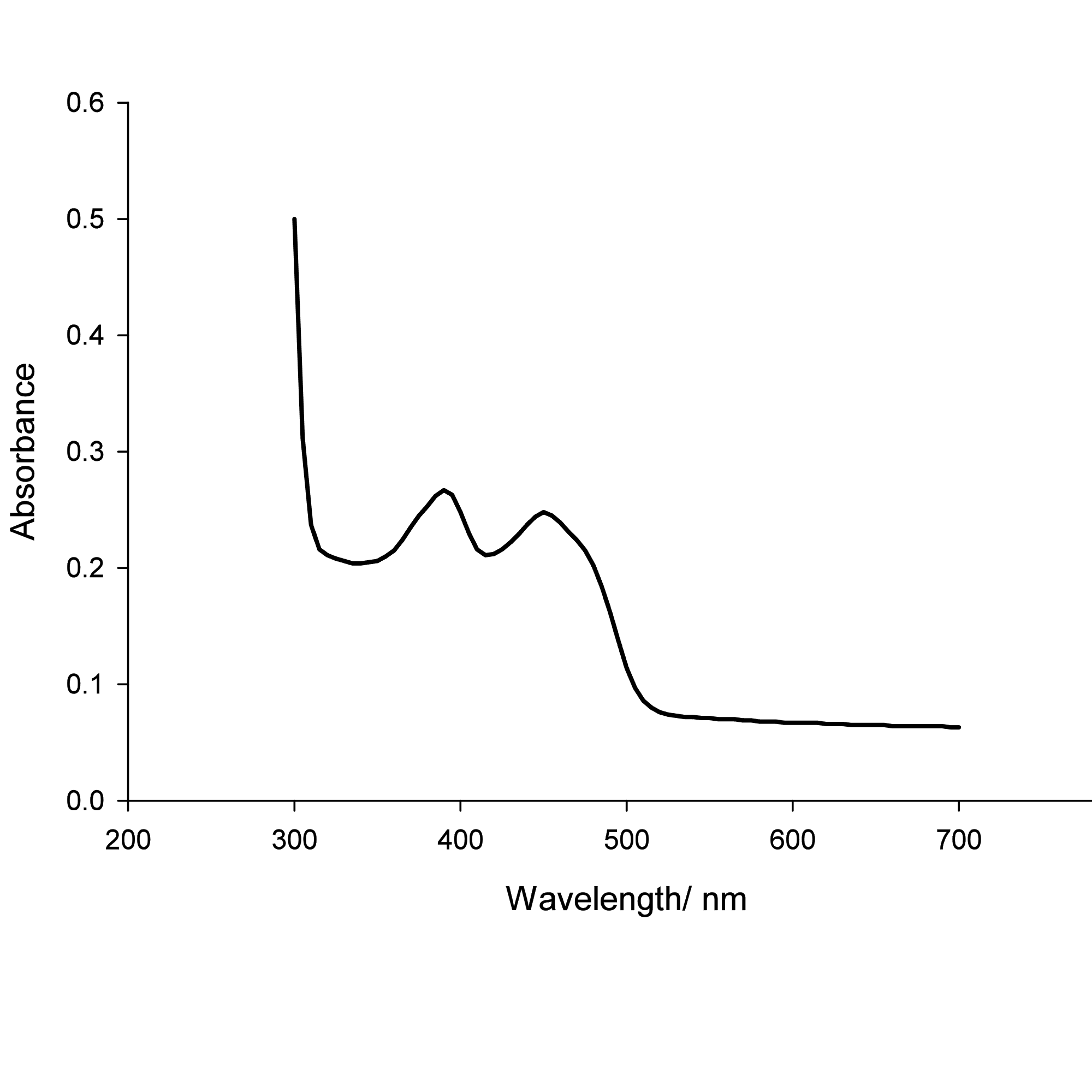


**N202**

**N167**


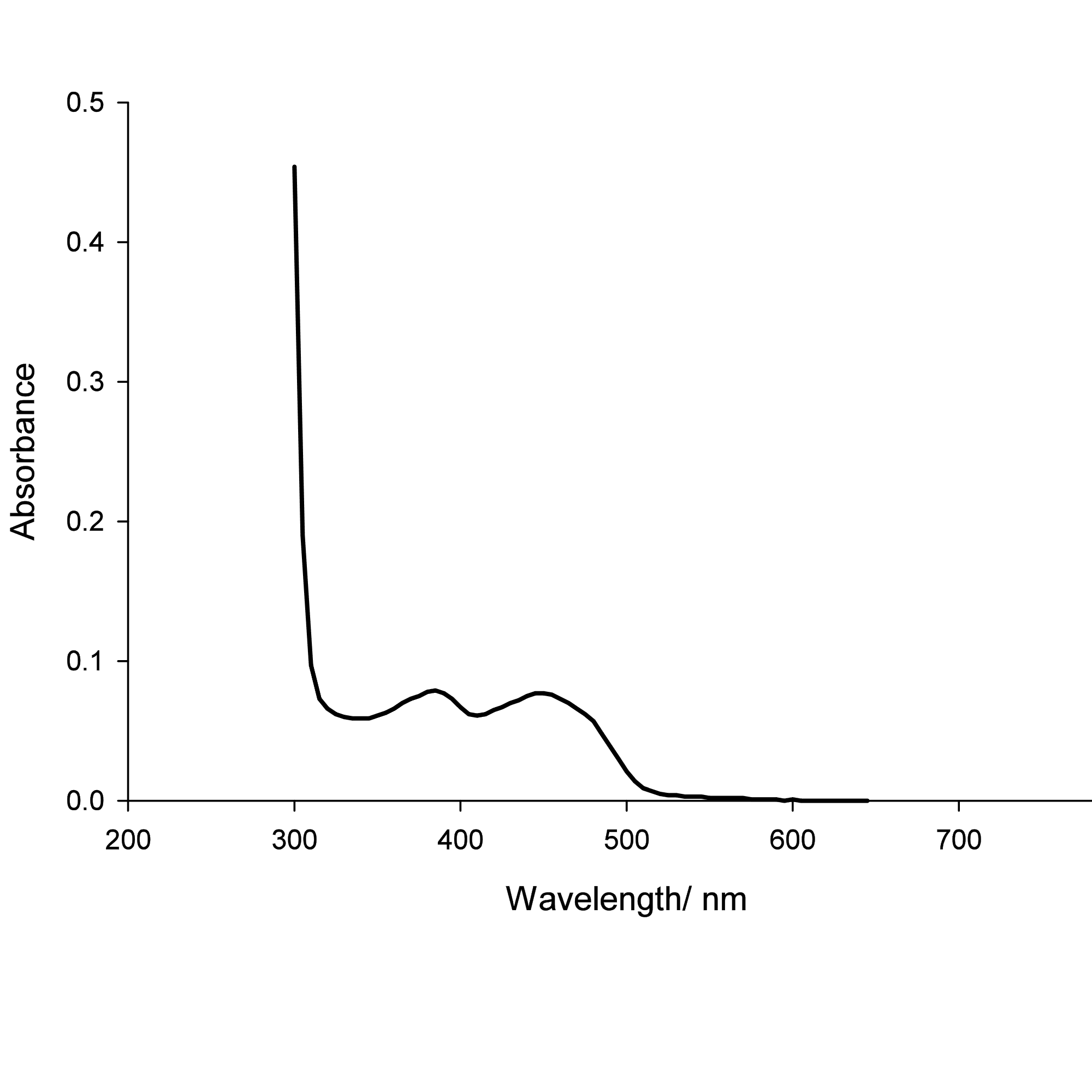

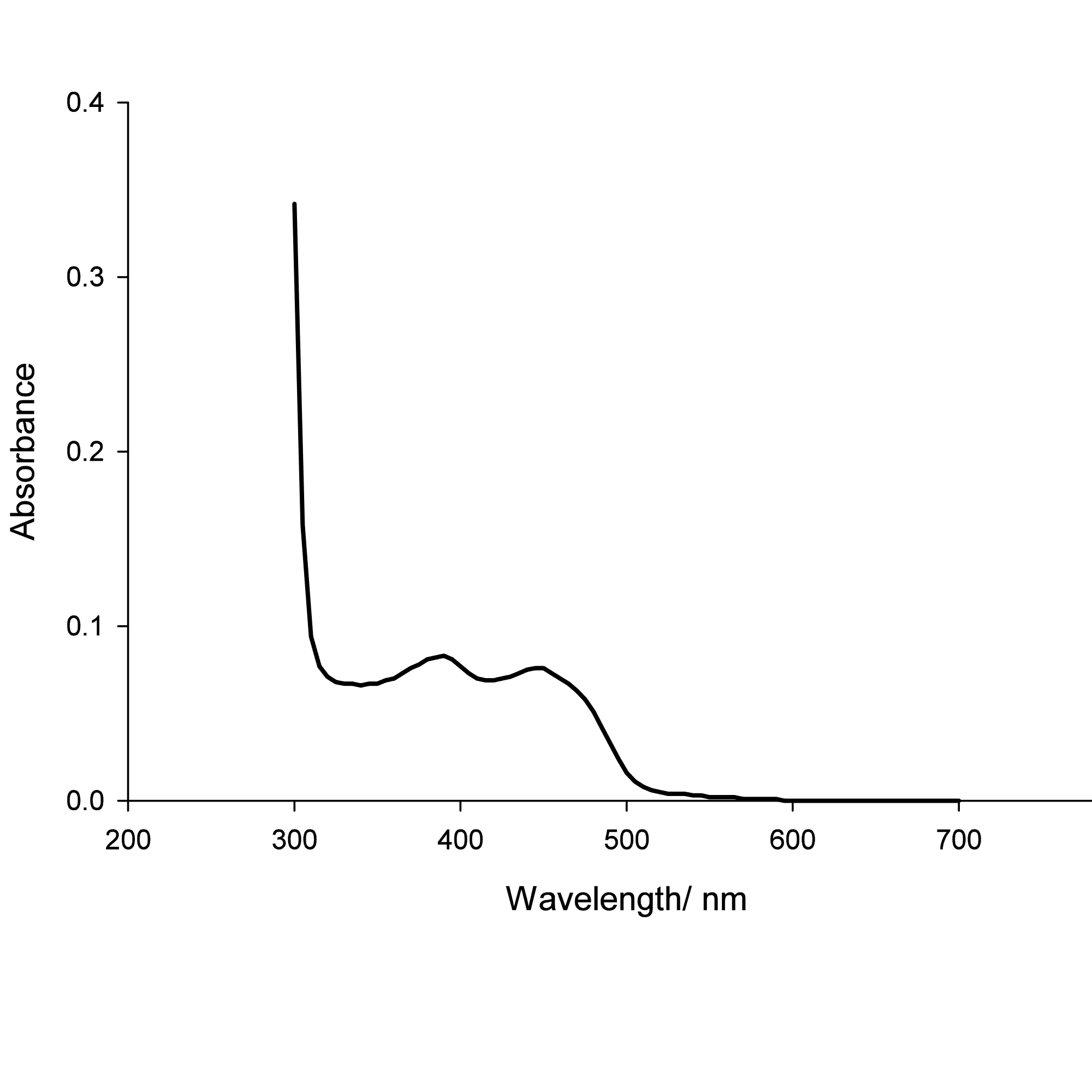


***Sc*POx**

**N284**


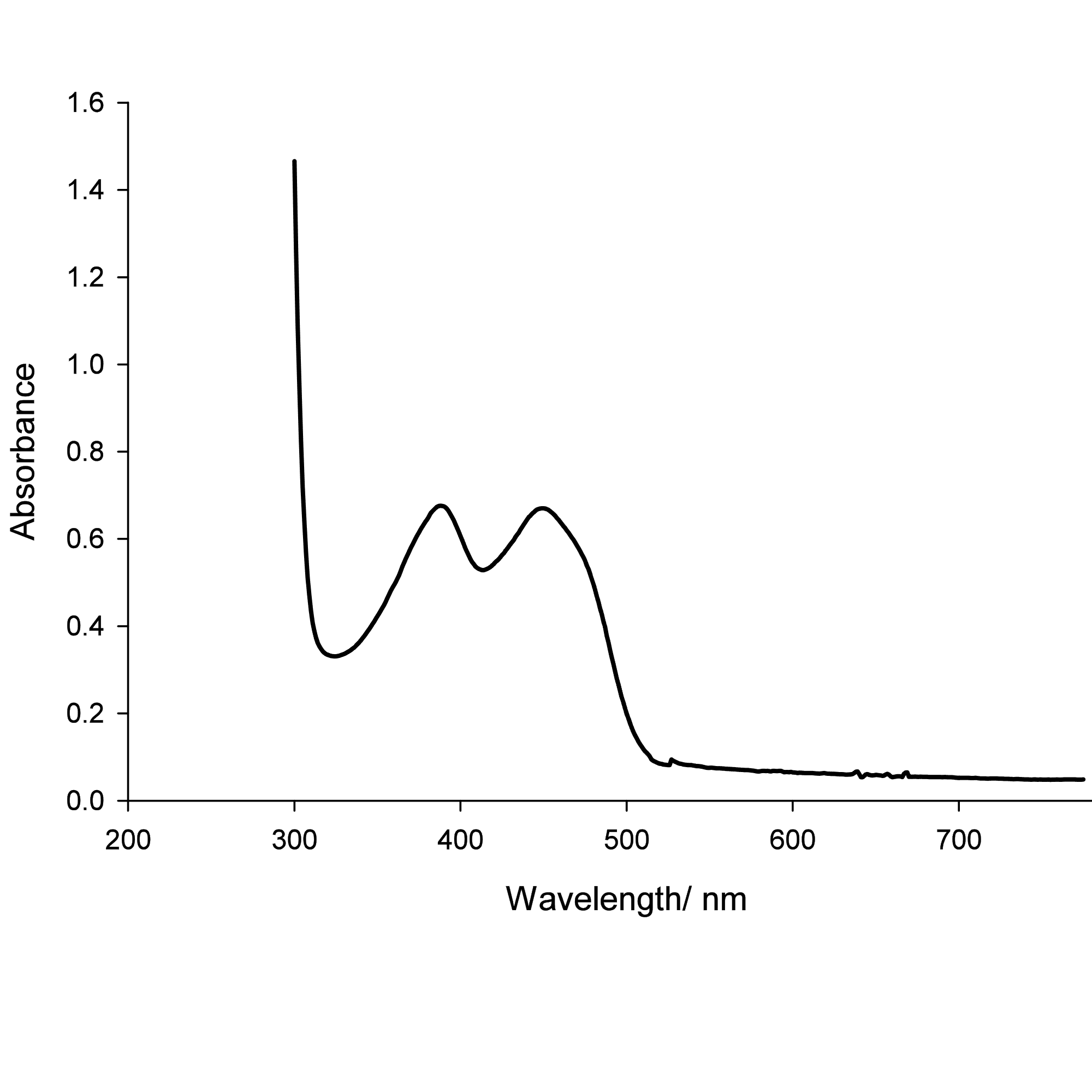
 **
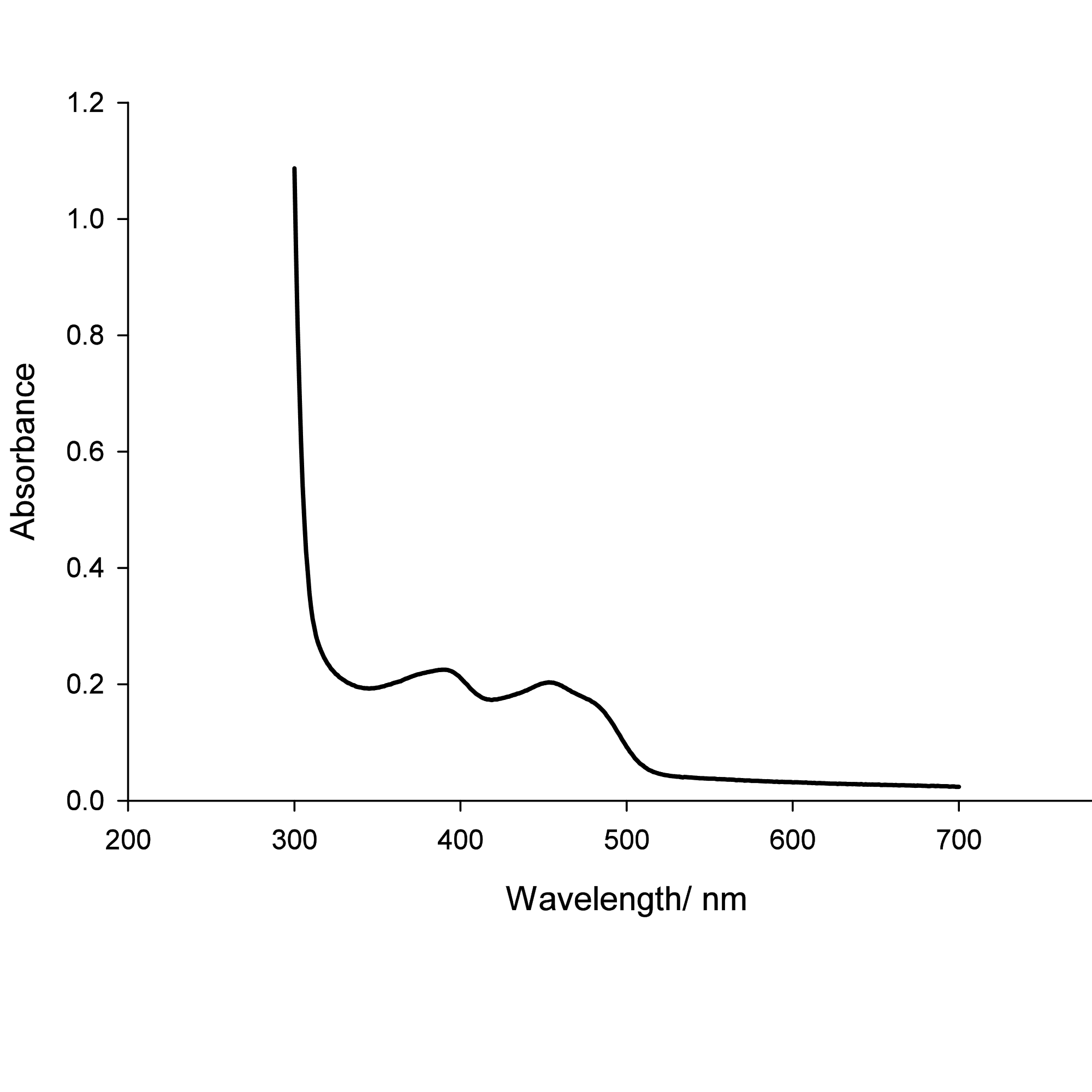
**

**N383**

**N327**

**
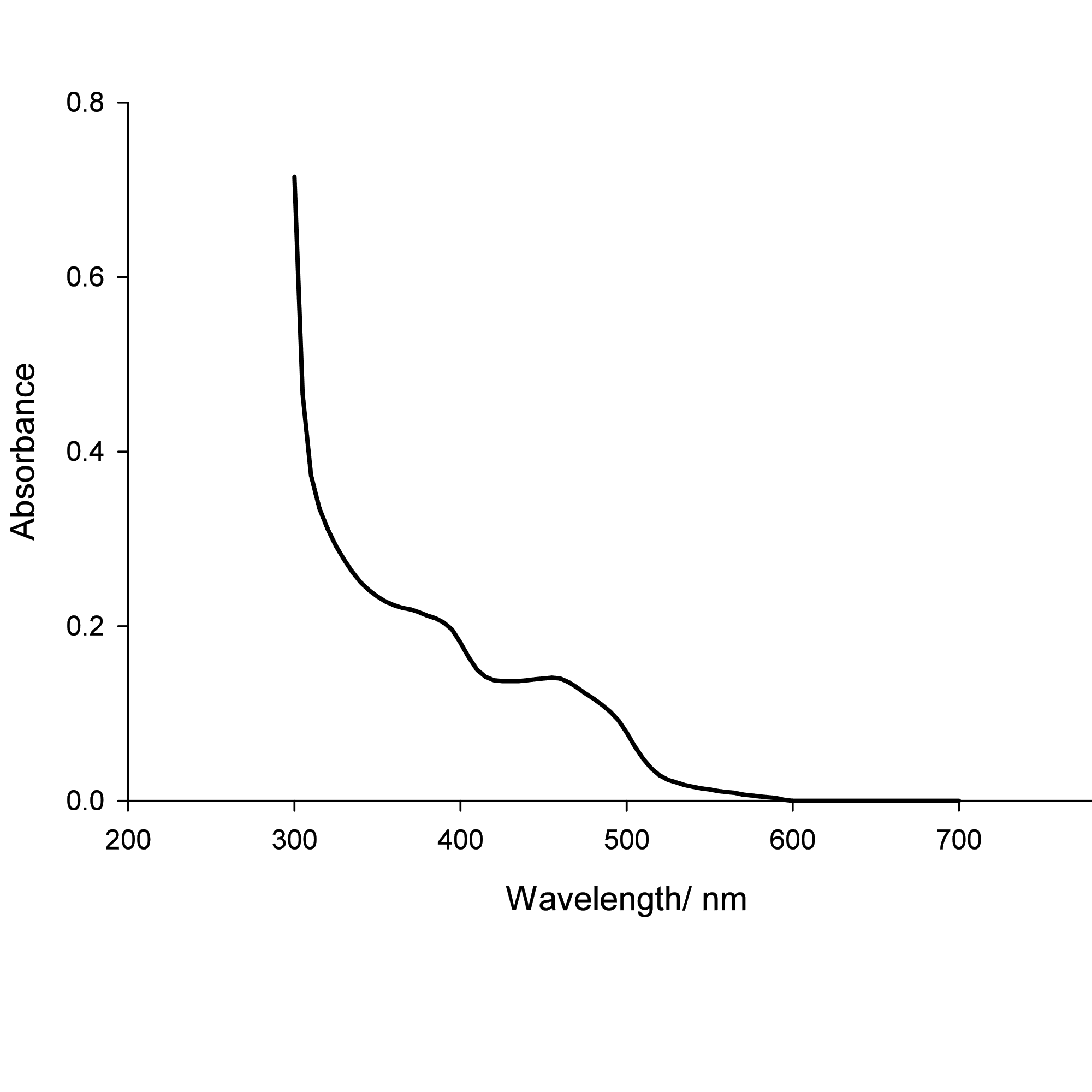
**
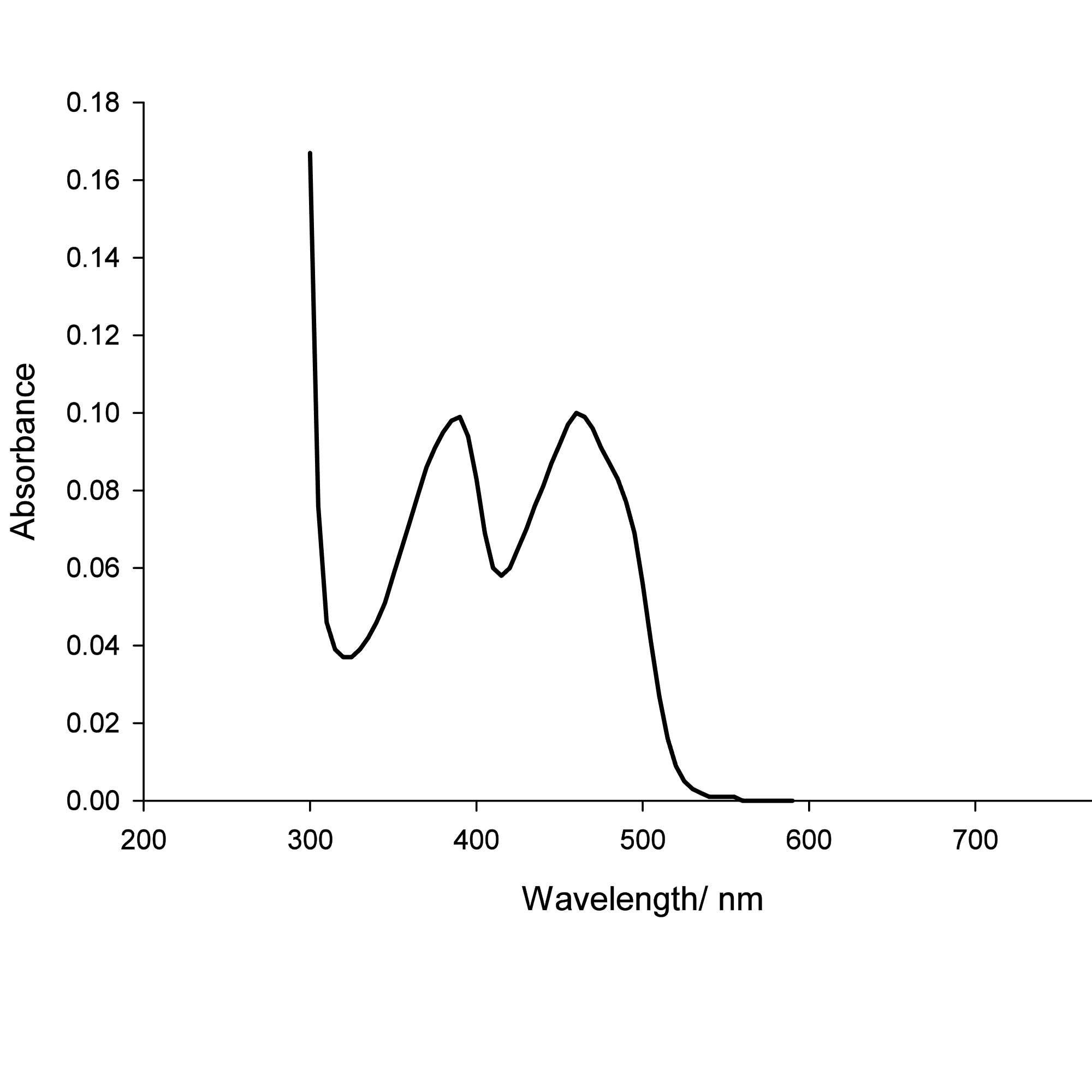


***Ps*POx**


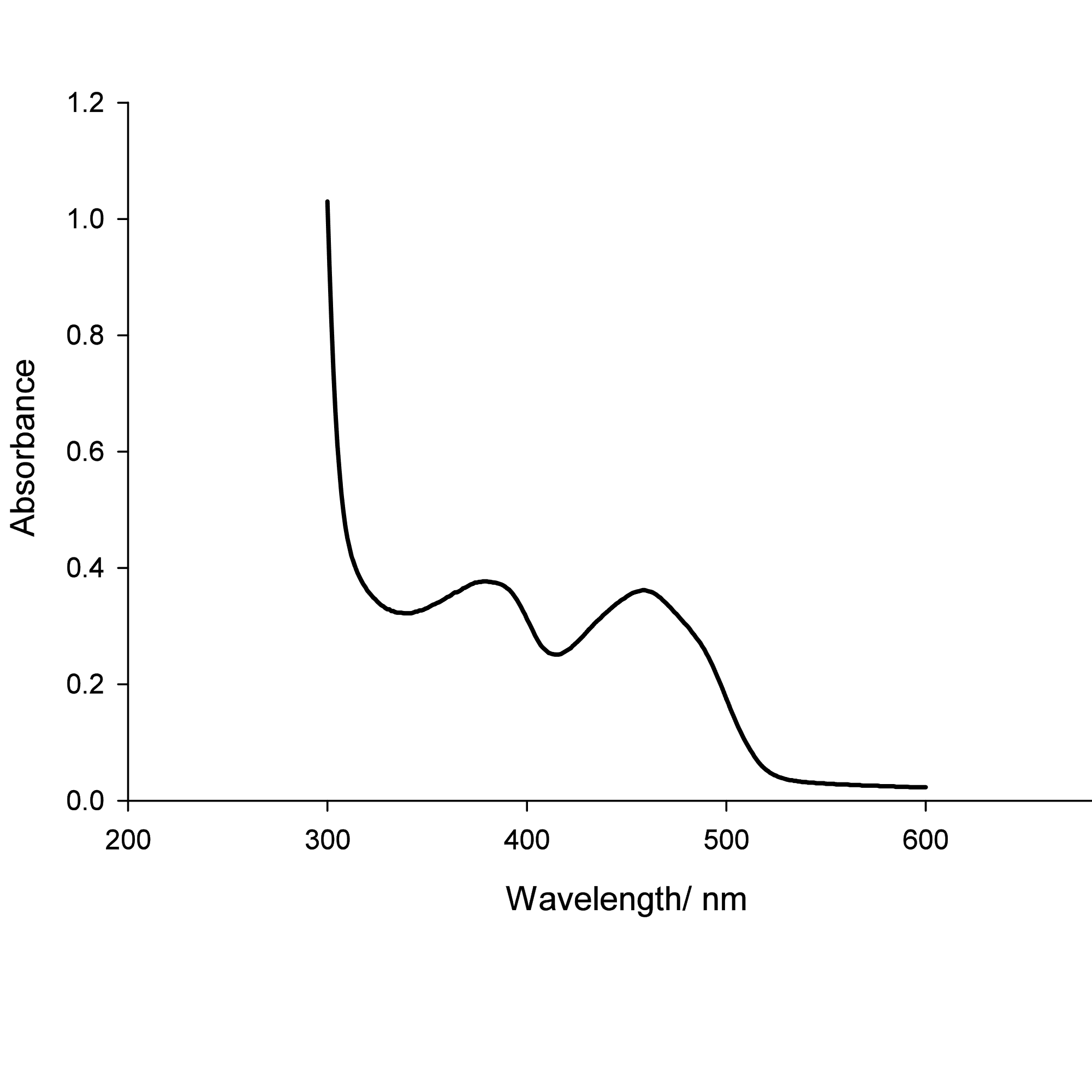


**Figure S4.** UV/Vis absorption spectra (300-700 nm) after reconstitution with FAD for N35, N67, N167, N202, *Sc*POx (3), N284, N327, N383 and *Ps*POx (4, 5).

**Table S3.** Lambda maxima and associated extinction coefficient for the spectra (Figure S4) for N35, N67, N167, N202, *Sc*POx (3), N284, N327, N383 and *Ps*POx (4, 5).

| **Enzyme** | **Lambda maximum/nm** | **Associated extinction coefficient/M^-1^ cm^-1^** |
| --- | --- | --- |
| N35 | 390 | 5070 |
|  | 450 | 4670 |
| N67 | 390 | 6340 |
|  | 450 | 5900 |
| N167 | 385 | 2090 |
|  | 450 | 2040 |
| N202 | 390 | 1950 |
|  | 450 | 1780 |
| *Sc*POx (3) | 390 | 9500 |
|  | 455 | 9440 |
| N284 | 390 | 3030 |
|  | 450 | 2730 |
| N327 | 390 | 3960 |
|  | 455 | 2740 |
| N383 | 390 | 2630 |
|  | 460 | 2660 |
| *Ps*POx (4, 5) | 380 | 11,100 |
|  | 460 | 10,620 |

**N202**

**N67**


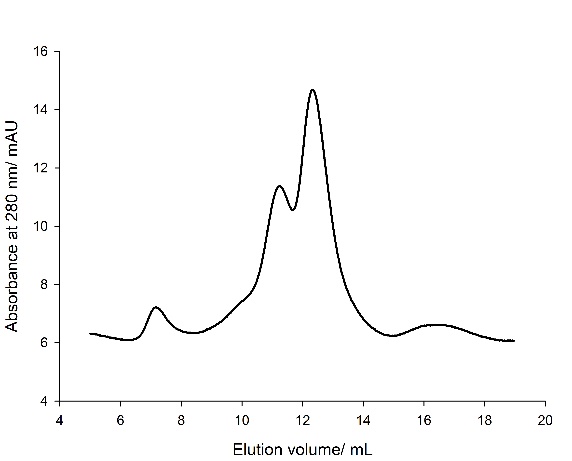

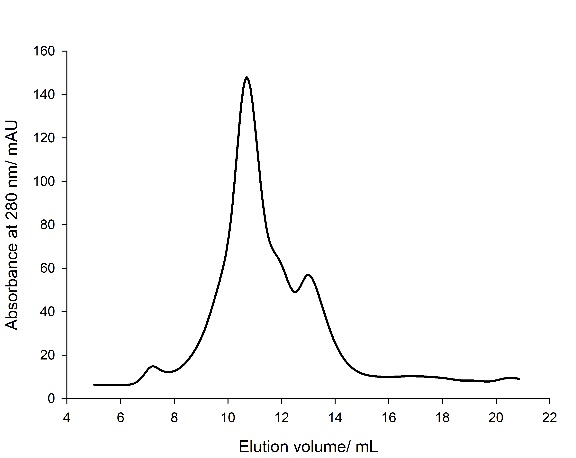


**Figure S5.** The peak indicated with a black dot represents the active fraction of the enzyme samples N67 and N202 tested with a routine DCIP assay using 0.2 mM homoorientin, as described in the Materials and Methods section

**Table S4.** Average pairwise sequence identity between extant and ancestral primary sequences belonging to the POx and CGOx sequence space and the sequence of *Tm*POx (1) and *Ps*POx (4, 5).

| **Enzyme** | **Pairwise identity to** | |
| --- | --- | --- |
|  | ***Ps*POx/%** | ***Tm*POx/%** |
| *Tm*POx (1) | 26.0 | - |
| N1 | 29.0 | 38.8 |
| N6 | 31.5 | 40.1 |
| N12 | 36.3 | 43.0 |
| N22 | 37.2 | 43.5 |
| N29 | 35.8 | 39.4 |
| N34 | 35.4 | 39.4 |
| *Ka*POx(2) | 34.9 | 38.6 |
| N35 | 48.3 | 28.9 |
| N67 | 46.2 | 24.4 |
| N167 | 43.3 | 26.0 |
| N202 | 41.1 | 25.5 |
| *Sc*POx (3) | 37.7 | 24.2 |
| N284 | 56.8 | 28.4 |
| N327 | 74.9 | 27.6 |
| N383 | 83.7 | 26.6 |
| *Ps*POx (4, 5) | - | 25.9 |
| *Mt*CarA (6) | 71.9 | 26.2 |


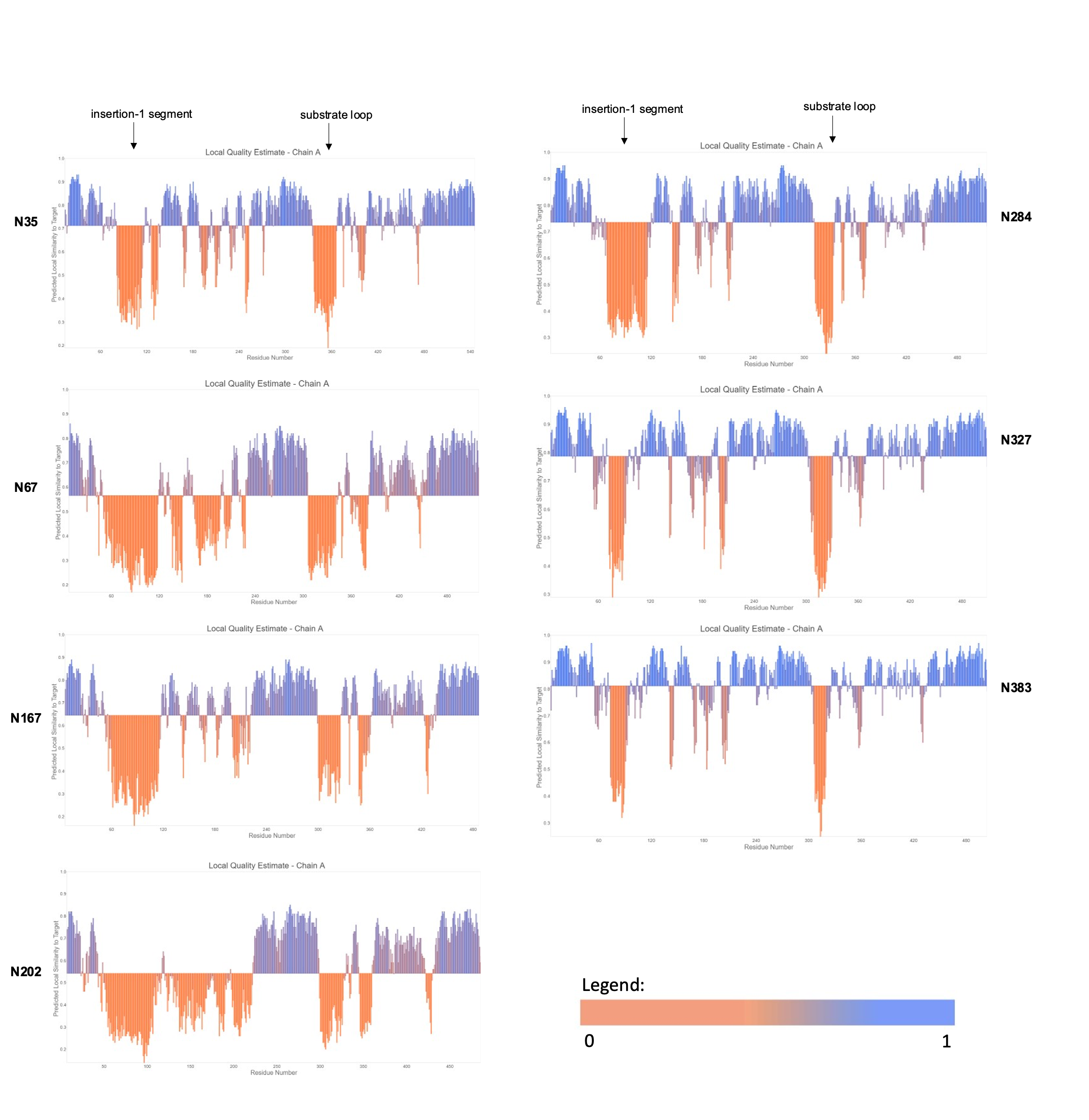


**
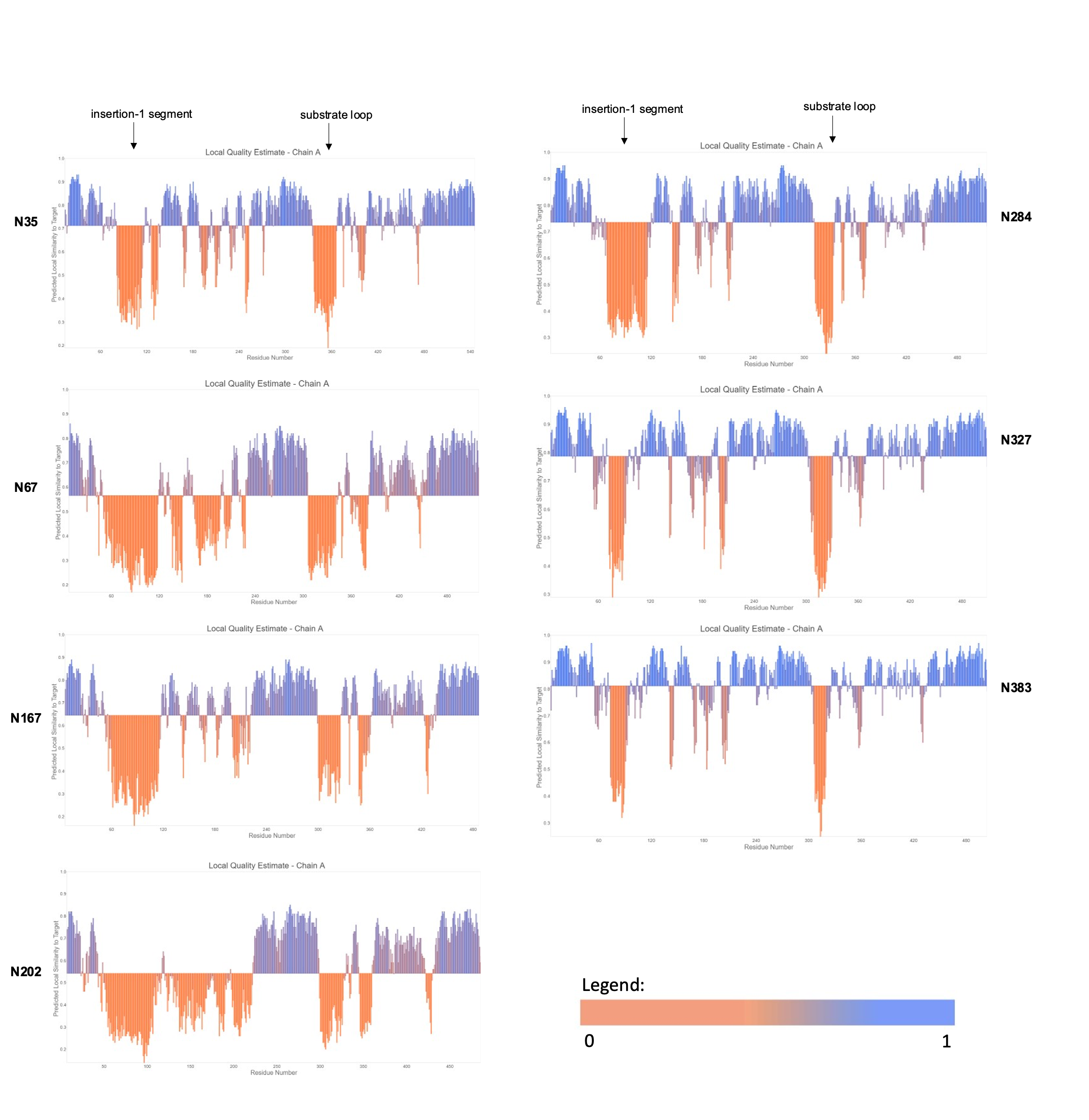
**

**Figure S6.** Predicted local similarity statistical data of ancestral structural models to the target crystal structure of *Ps*POx. The spatial fidelity of each position of amino acids is scored by the QMEANDisCo function (7). The local confidence is coloured based on the colour scale, where the colour on the left side of the bar represents lowest confidence (0) and the colour on the right side of the bar represents highest confidence (1). Approximate positions of the insertion-1 segment and the substrate loop are indicated in the figure.

N-terminal domain

**TmPOx**  **MSTSSSDPFF** **NFAKSSFRSA** **AAQKASASSL** **PPLPGPDKKV** **PGMDIKYDVV** **IVGSGPIGCT** 60

**PcPOx**  **MFLD-TTPF-** **-------R--** **----------** **----------** **--ADEPYDVF** **IAGSGPIGAT** 27

**KaPOx**  **MITR------** **----------** **----------** **----------** **-----YTDTL** **VVGSGPVGAT** 19

**N35**  **MTSHESTSL-** **----QCCK--** **----------** **----------** **--AAERTDVL** **IVGSGPVGSA** 31

**N67**  **MSH-------** **----------** **----------** **----------** **-----NPTIA** **IVGSGPIGSA** 18

**N167**  **MTST------** **----------** **----------** **----------** **---QSTADVL** **IVGSGIMGAA** 21

**N202**  **MTDT------** **----------** **----------** **----------** **---PRT-DVL** **IVGSGIMGSV** 20

**ScPOx**  **MTHT------** **----------** **----------** **----------** **---PRT-DVL** **IVGSGIMGSL** 20

**N284**  **MTT-------** **----------** **----------** **----------** **-----EYDVV** **VVGSGPVGSA** 18

**N327**  **M-TR------** **-------R--** **----------** **----------** **--YPSSVDVA** **IVGSGPAGAA** 22

**N383**  **MSAR------** **-------R--** **----------** **----------** **--YPASVDVA** **IVGSGPTGAA** 23

**PsPOx**  **MSGH------** **-------R--** **----------** **----------** **--YPAAVDVA** **IVGSGPTASA** 23

**MtCarA** **MSTR------** **-------V--** **----------** **----------** **--YPAQVDVA** **IVGSGPAGAT** 23

**TmPOx**  **YARELVGA--** **GYKVAMFDIG** **EIDSG-----** **----------** **-------LKI** **GAHKKNTVEY** 96

**PcPOx**  **FAKLCVDA--** **NLRVCMVEIG** **AADSFTSKPM** **KGDPNAPRSV** **QFGPGQVPIP** **GYHKKNEIEY** 85

**KaPOx**  **FARTLVES--** **GREVLMVDAG** **AQLS------** **----------** **-------PRP** **GEHLKNAYIY** 54

**N35**  **FARLIADARP** **SASILMVEAG** **PQLT------** **----------** **-------DPP** **GMNVRNIPDP** 68

**N67**  **YARLLLESLP** **DARVVMFEAG** **PQLT------** **----------** **-------DRP** **GESVRNIADP** 55

**N167**  **VARQVREARP** **GARILMVDAG** **PVIG------** **----------** **-------SVP** **GQHLHDSPDE** 58

**N202**  **VARLLRETDP** **ALRITMVDGG** **SAIG------** **----------** **-------SAP** **GLHLHDVDDP** 57

**ScPOx**  **VARLLRRSDP** **ALHITMADGG** **SPIG------** **----------** **-------GVP** **GRHLHDLDDP** 57

**N284**  **FARRVHDAAP** **SARVLMVEAG** **PRLT------** **----------** **-------DPP** **GAHVRNLPDA** 55

**N327**  **YARILSEQAP** **EATIAVFEVG** **PTVS------** **----------** **-------DPP** **GAHVKNIADP** 59

**N383**  **YARILSEQAP** **DATIAMFEVG** **PTVS------** **----------** **-------DPP** **GAHVKNIADP** 60

**PsPOx**  **YARILSEEAP** **GATIAMFEVG** **PTVS------** **----------** **-------NPP** **GAHVKNIEDP** 60

**MtCarA** **YARILSERAS** **SATIAMFEVG** **PTVS------** **----------** **-------DPP** **GAHVKNIADA** 60

Arm domain

**TmPOx**  **QKNIDKFVNV** **IQGQ------** **----LMSVSV** **P--------V** **NTLVVDTLSP** **T---SWQAST** 135

**PcPOx**  **QKDIDRFVNV** **IKGA------** **----LSTCSI** **P--------T** **SNNHIATLDP** **SVV-SNSLDK** 126

**KaPOx**  **QHNTNLFASI** **IRGH------** **----LHLLSV** **P--------T** **SARAELAVDP** **AAMAELGSNR** 96

**N35**  **EERAAAQAR-** **SQGPNSATAQ** **AGEARAVVGV** **PALGDRPAGA** **AAEGTITARP** **G--------T** 119

**N67**  **DEKARAREM-** **SQGP-----Q** **AGAFRESLGI** **P------AGT** **VVEGMFTARQ** **G--------T** 95

**N167**  **EIWARYNQRV** **ASGV------** **-QSMYVGAAT** **T-------AD** **VGGSLASVEP** **G--------M** 96

**N202**  **VLWSRYNEQV** **GTGI------** **-QGMYTGAEV** **V-------RE** **VADSLTGLTP** **G--------M** 95

**ScPOx**  **DLWSRYNEKV** **ATGI------** **-QGMYTGAEV** **V-------RD** **VAGSLPDLTP** **G--------M** 95

**N284**  **EERAAAQAR-** **SQGPNPATAQ** **VSAASATVSA** **PA----PVGA** **SREGAVTARP** **G--------T** 102

**N327**  **AERARAQRR-** **SEGP------** **-SAASPTVTN** **P-------GA** **AKDGARRARP** **G--------T** 96

**N383**  **AARAAAQRR-** **SEGP------** **-GAGAATVSS** **P-------GA** **VKSGQRRARP** **G--------T** 97

**PsPOx**  **DSRSLAQRA-** **SEGP------** **-GAGAATVNS** **P-------GA** **VKSGERRARP** **G--------T** 97

**MtCarA** **DERAHAQRR-** **SEGP------** **-HAREDDDRV** **G-------GI** **VKSAQRRARP** **G--------T** 97

Insertion-1 segment

Arm domain

**TmPOx**  **FFVRN-GSNP** **EQDPLRNLSG** **QAVTRVVGGM** **STHWTCATPR** **F--------D** **REQRPLLVKD** 186

**PcPOx**  **PFISL-GKNP** **AQNPFVNLGA** **EAVTRGVGGM** **STHWTCATPE** **FFAPADFNAP** **HRERPKLSTD** 185

**KaPOx**  **SSARN-AENP** **DQDPYRNLSA** **AAACYAVGGM** **GTHWTGATPR** **HH-------P** **VLERYDGI--** 146

**N35**  **FLIDPGGSAA** **DGQPG-AMPA** **AAMSTCVGGM** **GAHWTCATPR** **P--------A** **GSERIPFI--** 168

**N67**  **HLLDF-GGEG** **SAHAP-TFPA** **AAAATNVGGQ** **GAHWTCATPR** **P--------A** **FSEKIPFI--** 143

**N167**  **YHLSALGEDA** **AA-----MPA** **AALAWNVGGM** **GVHWTAACPW** **P--------W** **GSEVFDFI--** 141

**N202**  **FHALAFGEDA** **EA-----MPA** **TALAWNAGGM** **GVHWTAATPW** **P--------A** **GDEVFDFG--** 140

**ScPOx**  **FHALAFGEDA** **EA-----MPQ** **AALAWNAGGM** **GVHWTAATPW** **P--------A** **GDEVFDFG--** 140

**N284**  **FLIRP-GSAA** **DGQTG--MPA** **AAMSSNVGGM** **GAHWTCACPR** **P--------G** **DSERIPFI--** 149

**N327**  **FLLPD-GWRV** **EGEDG--LPA** **AAMSSNVGGM** **GAHWTGACPR** **P--------G** **GSERIAFL--** 143

**N383**  **FLLED-GYQV** **DGEDG--LPV** **AAMSSNVGGM** **AAHWTGACPR** **P--------G** **GSERIAFL--** 144

**PsPOx**  **YLLQD-GYAF** **PGEDG--MPV** **AAMSSNVGGM** **AAHWTAACPR** **P--------G** **GKERIPFL--** 144

**MtCarA** **YLLES-GYQA** **DGEDG--LPV** **AAFSSNVGGM** **AAHWTGACPR** **P--------N** **DSERIGFL--** 144

Flavinylation

motif

**TmPOx**  **DADADDAEWD** **RLYTKAESYF** **QTGTDQFKES** **IRHNLVLNKL** **TEEYKGQ---** **-RDFQQIPLA** 242

**PcPOx**  **AAED-ARIWK** **DLYAQAKEII** **GTSTTEFDHS** **IRHNLVLRKY** **NDIFQKE-NV** **IREFSPLPLA** 243

**KaPOx**  **---S-DQEWD** **GLYGEAERLL** **RVSAREFDFS** **IRQHLVTEAL** **RREFSEL-PD** **GYQVQSLPLA** 201

**N35**  **---P-AAEWD** **AALDEAERLL** **HVTQSAFPDS** **PQGEAIREAL** **REEFDGALPE** **GRKVQPLPLA** 224

**N67**  **---A-DDEWD** **DLIATAEGLL** **HVQSAAFADS** **AVGGAIRSLL** **EEEFGAELPE** **GYGPSTLPVA** 199

**N167**  **---P-AAEWD** **ADLDTAQRLL** **RVHPSPFGPT** **EAGDAVLEAL** **DEVFGPVSAP** **GRHPQPMPMA** 197

**N202**  **---D-PARWA** **ADLDTARRLL** **AVTPAAIGPT** **EVGRLVLDVL** **RRRYDGVGPD** **DRRPQPMPMA** 196

**ScPOx**  **---D-PDAWA** **ADLDTARRLL** **AVTPAPIGPT** **KVGELVLDVL** **RRRYGGTGPA** **DRAPQPMPMA** 196

**N284**  **---P-AAELD** **AALAEAERLL** **HVTQDAFPDT** **PLGDEVRRAL** **GEVFDEGRPP** **DRRVQPMPLA** 205

**N327**  **---P---DLD** **ELLDEAERLL** **GVSADAFADA** **PFADEVRARL** **GAALDEGRAP** **DRRVQPMPLA** 197

**N383**  **---P---DLD** **ELLDEAERLL** **GVTTDAFDGA** **PFGDLVRERL** **AAAVDEGREP** **ETRVQRMPLA** 198

**PsPOx**  **---P---DLE** **ELLNDADRLL** **GVTTHAFDGA** **PFSDLVRERL** **AAVVDQGRTP** **AFRVQPMPLA** 198

**MtCarA** **---DETGELD** **ELLSEGERLL** **GVTTDAFDAS** **PYAGIVRERL** **AAVEDAHRDA** **DERVQRMPLA** 201

**TmPOx**  **ATRRS--PTF** **VEWSSANTVF** **D-LQNRPNTD** **APEERFNLFP** **AVACERVVRN** **----ALNSEI** 295

**PcPOx**  **CHRLTD-PDY** **VEWHATDRIL** **EELF---TDP** **VKRGRFTLLT** **NHRCTKLVFK** **HYRPGEENEV** 299

**KaPOx**  **ARRRRDNPRM** **VHWTGVDTVL** **GDLA------** **DGHPLFSLLP** **QHLCTRLVLD** **----RDGTRI** 251

**N35**  **ATPRPDGS--** **LRWTGTDTVL** **GPLAD--DAS** **SGDPRFTLRP** **ETLCRRLVLE** **-----DGDRV** 275

**N67**  **GDPQPDGS--** **MRWAGADVVL** **GPLID--PGS** **PLSERFELRD** **LTLVRRVEHD** **-----GD-RV** 249

**N167**  **VTPSPSGP--** **LRRTGPNRIF** **PPIA-----D** **GGDPAFTLLP** **GTLCTRLLHD** **-----GG-RV** 244

**N202**  **VTATASGP--** **MPRTAPGTIF** **PAIA-----T** **GGDPAFTLLT** **GTLATSLLVS** **-----GG-RV** 243

**ScPOx**  **VTPTPSGP--** **MPRTAPGTIF** **PPLA-----Q** **GGDPAFTLLT** **GTLVTALVRD** **-----AG-RV** 243

**N284**  **VTPRPDGS--** **PRWSGTDTVL** **G------DAA** **SSDPRFELRP** **ETLCRRVLLE** **-----DG-RV** 251

**N327**  **VHRRDDGR--** **LVWSGSDVVF** **G------DAT** **RANPNFTLFD** **ESLVTRVLVE** **-----DG-RA** 243

**N383**  **VHRRDDGR--** **LVWSGSDVVL** **G------DVT** **RANPNFTLFD** **ESLVTRVLVE** **-----DG-RA** 244

**PsPOx**  **VHRRQDGA--** **LVWSGSDVVM** **G------EAT** **RDNPQFELFD** **ESLVTRVLVE** **-----DG-TA** 244

**MtCarA** **VHRRDDGP--** **LVWSGSDVVL** **G------DIT** **RGNPNFTLFD** **ESLVTRVLVE** **-----DG-RA** 247

**TmPOx**  **ESLHIHDL--** **------ISGD** **RFEIKADVYV** **LTAGAVHNTQ** **LLVNSGFGQL** **GRPN------** 341

**PcPOx**  **DYALVEDLLP** **HMQNPGNPAS** **VKKIYARSYV** **VACGAVATAQ** **VLANSHI---** **-PPDDVVIPF** 355

**KaPOx**  **AYAEVRDL--** **------NRSE** **TVRVVADNYV** **VAAGAVLAPQ** **LLHASGI---** **-RP-------** 292

**N35**  **TGAEVRDL--** **------RTGE** **TSTVRADVVV** **VAADALRTPQ** **LLWASGI---** **-RP-------** 316

**N67**  **TGVTVEDL--** **------RTRE** **TSFVPADLVV** **VAADAFRSPQ** **LLWASGI---** **-RP-------** 290

**N167**  **TGARVRDI--** **------ATGE** **ERTIEASVVV** **VCADTLRTPQ** **LLFASGI---** **-RP-------** 285

**N202**  **TGARLRRV--** **------ADGA** **ESELRADTVV** **VCADALRTPQ** **LLFASGI---** **-RP-------** 284

**ScPOx**  **TGARLRRV--** **------ADGT** **ESELSADTVV** **VCADALRTPQ** **LLYASGI---** **-RP-------** 284

**N284**  **TGVELRDL--** **------RTGE** **VHTVRARAVV** **VAADALRTPQ** **LLWASGI---** **-RP-------** 292

**N327**  **AGVRVRDL--** **------RTGE** **EHEVRARFVV** **VAADALRTPQ** **LLWASGI---** **-RP-------** 284

**N383**  **AGVEVRDR--** **------RTGE** **THTVRARFVV** **VAADALRTPQ** **LLWASGI---** **-RP-------** 285

**PsPOx**  **AGVEVQDR--** **------RSGD** **TYQVAARYVV** **VGADALRTPQ** **LLWASGI---** **-RP-------** 285

**MtCarA** **AGVVVTDV--** **------RTGE** **RRDVRARFVV** **VAADALRTPQ** **LLWASGI---** **-RP-------** 288

Head domain

**TmPOx**  **----------** **---PANPPEL** **LPSLGSYITE** **QSLVFCQTVM** **STEL---IDS** **VKSDMTIRGT** 385

**PcPOx**  **PGGEKGSGGG** **ERDATIPTPL** **MPMLGKYITE** **QPMTFCQVVL** **DSSL---MEV** **VRN------P** 406

**KaPOx**  **----------** **----------** **-AALGRYLTE** **HPMAFCQVIL** **LKDL---VEQ** **ARTDQRFG--** 326

**N35**  **----------** **----------** **-PALGRYLTE** **HPLVFSAVAL** **DPEL---VSA** **AAADAPVS--** 350

**N67**  **----------** **----------** **-RALGHYLTE** **HPVVISTVAL** **DAER---MSR** **FATEEDLD--** 324

**N167**  **----------** **----------** **-PALGRHLNE** **HAFLTGRVLA** **DLER---LGV** **DLSSLPA---** 318

**N202**  **----------** **----------** **-EALGRYLNE** **HAFITARVLL** **DLDR---FGI** **GLDALPL---** 317

**ScPOx**  **----------** **----------** **-EALGRHLNE** **HAFVTARVLL** **DLDR---FGL** **DPDALPL---** 317

**N284**  **----------** **----------** **-PALGRYLND** **HPQVVSAVRL** **DDEL---VAA** **PAAHAETS--** 326

**N327**  **----------** **----------** **-PALGRYLND** **QPQVVFAVRL** **RDVAPAPASA** **PAAAADSS--** 321

**N383**  **----------** **----------** **-DALGRYLND** **QAQVVFATRI** **RDV------A** **PAAAADAA--** 316

**PsPOx**  **----------** **----------** **-DALGRYLND** **QAQVVFASRL** **RDV------Q** **P-EDAPAA--** 315

**MtCarA** **----------** **----------** **-DALGRYLND** **QAQIVFAVRM** **RDF------T** **PVVDADGV--** 319

Head domain

**TmPOx**  **PGELTYSVTY** **TPGASTNKHP** **DWWNEKVKNH** **MMQHQEDPLP** **IPFEDPEPQV** **TTLFQ-PSHP** 444

**PcPOx**  **P---------** **WPGL------** **DWWKEKVARH** **VEAFPNDPIP** **IPFRDPEPQV** **TIKFT-EEHP** 450 **KaPOx**  **----------** **----------** **----GQVARH** **TTLFPDDDLP** **IPVDDPEPNV** **WIPVS-EGRP** 361

**N35**  **----------** **----------** **----AEAAST** **A--------A** **GPADPLTAVV** **WIPFSEEAHP** 378

**N67**  **----------** **----------** **----AELARR** **A--------T** **NPADPVAAVN** **RIPFSEPDHP** 352

**N167**  **----------** **----------** **----------** **---------P** **REGEWAVGSY** **WLPHSGAAQP** 339

**N202**  **----------** **----------** **----------** **---------P** **RVGEFSTDSL** **WLPQNGAAQP** 338

**ScPOx**  **----------** **----------** **----------** **---------P** **RPGEFSTDSL** **WLPCNGPSQP** 338

**N284**  **----------** **----------** **----AD----** **----------** **GARDLLTGVS** **WVPFSDERHP** 348

**N327**  **----------** **----------** **----AD----** **----------** **GALTAQSGVS** **WVPFTDE-HP** 342

**N383**  **----------** **----------** **----AA----** **----------** **GALSEQSGVS** **WVPFTDE-MP** 337

**PsPOx**  **----------** **----------** **----AN----** **----------** **GALSEQSGVA** **WVPYTDE-AP** 336

**MtCarA** **----------** **----------** **----PQ----** **----------** **TGLSEYTGVT** **WVPFTDD-MP** 340

Substrate loop

**TmPOx**  **WHTQIHRD--** **-AFSYGAVQQ** **SIDSR-LIVD** **WRFFGRTEPK** **EENKLWFSDK** **ITDAYNMPQP** 500

**PcPOx**  **WHVQIHRD--** **-AFSYGAVAE** **NMDTR-VIVD** **YRFFGYTEPQ** **EANELVFQQH** **YRDAYDMPQP** 506 **KaPOx**  **WHAQITRD--** **-AFHYGDVPP** **HVDGR-LIVD** **LRWFGIVEPR** **PDNRVTFSDT** **RTDVMGMPQP** 417

**N35**  **YHAQIMHMAT** **SPFPLDEDSA** **HAGN--PVVG** **MGWFVRKDPR** **AEDRVTFSDT** **ETDAYGMPQM** 436

**N67**  **FSLQVMYSET** **TPFPMDPDAP** **HANNRWGYVN** **MGYGMRKHPR** **FEDAVTFDDD** **EPDYRGFPNM** 412

**N167**  **FHGQIMDR--** **-PFVDEDGEP** **LAYS----VG** **LSLYVPTEVR** **AENRLEFSDT** **ETDAAGMPRM** 392

**N202**  **FHGQIMNR--** **-TYVDEDGRP** **LAHS----VG** **LSLYVPVESR** **PENRLVFSET** **ETDLAGMPRI** 391

**ScPOx**  **FHGQIMNR--** **-TYVDGAGRP** **LAHS----VG** **LSLYVPVESR** **PQNRLVFSPG** **ETDLAGLPRI** 391

**N284**  **FHGQVMQLDA** **SPVPLGDDDS** **ADPG--SVVG** **LGWFAAKDIR** **AEDRVEFSDT** **ETDAYGMPAM** 406

**N327**  **FHGQVMQLDA** **SPVPLADDDD** **PAPG--SIVG** **LGWFCAKDLQ** **ASDRVEFSDD** **ETDAYGMPAM** 400

**N383**  **FHGQIMQLDA** **SPVPLAEDDP** **VVPG--SIVG** **LGLFCAKDLQ** **RDDRVEFSDD** **EVDAYGMPAM** 395

**PsPOx**  **FHGQIMQLDA** **SPVPLADDDP** **IVPG--SIVG** **LGLFCAKDLQ** **REDRVAFDDD** **TRDSYGLPAM** 394

**MtCarA** **FHGQVMQLDA** **SPVKLADDDP** **AAPG--SIVG** **LGLFCAKDLQ** **ASDRVAFSDS** **DVDGYGMPAM** 398

Catalytic His

**TmPOx**  **TFDFRFPAGR** **TSKEAEDMMT** **DMCVMSAKIG** **GFLPGSLPQF** **MEPGLVLHLG** **GTHRMGFDEK** 560

**PcPOx**  **TFKFTM-SQD** **DRARARRMMD** **DMCNIALKIG** **GYLPGSEPQF** **MTPGLALHLA** **GTTRCGL-DT** 564 **KaPOx**  **TFEYAL-SPQ** **DAERQHAMMA** **EMMRAATALG** **GFLPGSEPRF** **TAPGLPLHIA** **GTIRMGD-DP** 475

**N35**  **TIHYEL-TER** **DQAEIERAKA** **DMRRAAEALG** **EFVPGGEPRL** **MPAGSSLHYQ** **GTVRMGPADD** 495

**N67**  **TIEYAL-TEA** **EQAEIAEATE** **RLRRAGNALG** **TFV--AEPRL** **MPNGSSLHYQ** **GTMRMGEADD** 469

**N167**  **TVHFSY-SDR** **DLALIERARA** **QQRQAAEALG** **DFDPERDSAL** **LPPGSSLHYT** **GTVRMGPADD** 451

**N202**  **GVEFGY-SDA** **DRALIGRALD** **EVRSLAEEFG** **PFDPATERAL** **LPPGSSLHLT** **GTVRAGAADD** 450

**ScPOx**  **RVEFGY-SET** **DRALIRRALD** **EVRSVAEEFG** **PFDPATESTV** **LPPGSSLHLT** **GTVRAGVTDD** 450

**N284**  **TIHYSL-TDR** **DRATIERAKE** **AVTRAAAALG** **EPLP-GEPRL** **LPAGSSLHYQ** **GTVRMGPADD** 464

**N327**  **RIHYTL-TER** **DHETIARARE** **AIVRAAKALG** **EPLD-DEPLT** **LPPGSSLHYQ** **GTVRMGETDD** 458

**N383**  **RIHYRL-TDR** **DHEVIDRARE** **EIVRLGKAVG** **EPLD-DRPFT** **MPLGASLHYQ** **GTTRMGETDD** 453

**PsPOx**  **RIHYRL-TER** **DHVVLDRARQ** **EIVRLGKAVG** **EPLD-ERPFV** **LPPGASLHYQ** **GTTRMGETDD** 452

**MtCarA** **QLHYTL-SDR** **DHATIDRAKA** **EIVRLGKAIG** **DPLD-DRPFV** **MPLGASLHYQ** **GTVRMGLADD** 456

Catalytic Asn

**TmPOx**  **EDNCCVNTDS** **RVFGFKNLFL** **GGCGNIPTAY** **GANPTLTAMS** **LAIKSCEYIK** **QNFTPSPFTS** 620

**PcPOx**  **QKTVG-NTHC** **KVHNFNNLYV** **GGNGVIETGF** **AANPTLTSIC** **YAIRASNDII** **AKF-------** 616 **KaPOx**  **QSSVV-DTDS** **RVWGLENLYL** **GGNGVIPTGT** **ACNPTLTSVA** **MALKAAHHLA** **GS-------R** 527

**N35**  **GTSVC-DPHS** **RVWGFENLYL** **GGNGVIPTAT** **ACNPTLTSVA** **LAVRAARALV** **AR--------** 546

**N67**  **GTSVA-DPWS** **RVWGYENLVV** **GGNALIPTAT** **AMNPTLMSVA** **IAVRGARKAA** **EE--------** 520

**N167**  **GTSVC-DPDG** **RVWGFDNLYL** **AGNGVIPTPV** **VCNSTLTGTV** **TAVRAARAVA** **AR--------** 502

**N202**  **GTSVC-DPDG** **RVWGFDNLYL** **AGNGVIPTAM** **AANVTLTGAV** **TAVRAARAVA** **AR--------** 501

**ScPOx**  **GTGVC-DPDG** **RVWGFDNLYL** **AGNGVVPTPM** **AANVTLTGAV** **TAVRTARAVT** **ART-------** 502

**N284**  **GTSVC-DPHS** **RVWGVENLYV** **GGNGVIPTAT** **ACNPTLTSVA** **LAVRAARAIA** **AR--------** 515

**N327**  **GTSVC-DPDS** **QVWGVPGLYV** **AGNGVIPTAT** **ACNPTLTSVA** **LAVRGARAIA** **AE--------** 509

**N383**  **GESVC-SPDS** **EVWGVPGLFV** **AGNGVIPTAT** **ACNPTLTSVA** **LAVRGARRIA** **AE--------** 504

**PsPOx**  **GESVC-SPDS** **QVWQVPGLFV** **AGNGVIPTAT** **ACNPTLTSVA** **LAVRGARKIA** **EEITSSLLMS** 511

**MtCarA** **GASVC-SPDS** **EVWGAPGLFV** **AGNGVIPTAT** **ACNPTLTGVA** **LAVRGARHIA** **DEITADLA--** 513

**TmPOx**  **EAQ-------** **--------** 623

**PcPOx**  **-GRHR-----** **--------** 620

**KaPOx**  **EARERRRTGA** **DEVLAVRS** 545

**N35**  **----------** **--------** 546

**N67**  **----------** **--------** 520

**N167**  **----------** **--------** 502

**N202**  **----------** **--------** 501

**ScPOx**  **----------** **--------** 502

**N284**  **----------** **--------** 515

**N327**  **----------** **--------** 509

**N383**  **----------** **--------** 504

**PsPOx**  **ESDNR-----** **-----LSK** 519

**MtCarA** **----------** **--------** 513

**Figure S7.** Multiple sequence alignment of the fungal enzymes *Tm*POx (1) and *Phanerochaete chrysosporium* POx (*Pc*POx) (8), present-day bacterial enzymes *Ka*POx (2), *Ps*POx (4, 5), *Sc*POx (3) and bacterial FAD-dependent *C*-glycoside 3-oxidase from *Microbacterium trichothecenolyticum* (*Mt*CarA) (6), together with ancestral sequences of N35, N67, N167, N202, N284, N327 and N383. Amino acids are coloured according to their physicochemical properties. Residues K55, R94, T129, Q297 and Q340 (from the *Ps*POx sequence) important to form hydrogen bonding with the aglycone moiety of *C*-glycoside are highlighted in yellow colour. Important structural and catalytical features are framed in black dotted or pink boxes.

**Table S5.** Number of mutations (amino acid exchanges) when comparing ancestors N35, N167, N202, N284, N327, and N383 to the extant enzymes *Ka*POx (2), *Sc*POx (3), *Ps*POx (4, 5) and *Mt*CarA (6) of the respective clades, as well as when comparing extant enzymes to each other. Additionally, the table includes the number of consensus mutations observed in the analysed ancestors. Ancestor N67 lacks any extant members for comparison.

**Clade I**

| **Enzyme** | N35 |
| --- | --- |
| *Ka*POx (2) | 291 |

**Clade III**

| **Enzyme** | N202 | N167 | N35 | Consensus N202-N167-N35 |
| --- | --- | --- | --- | --- |
| *Sc*POx (3) | 71 | 176 | 307 | 37 |

**Clade IV**

| **Enzyme** | N383 | N327 | N284 | N35 | Consensus N383-N327-N284-N35 |
| --- | --- | --- | --- | --- | --- |
| *Ps*POx (4, 5) | 87 | 133 | 207 | 273 | 38 |
| *Mt*CarA (6) | 108 | 141 | 208 | 268 | 36 |
|  |  |  |  |  |  |

**Extant enzymes**

| **Enzyme** | *Ka*POx | *Sc*POx | *Ps*POx | *Mt*CarA |
| --- | --- | --- | --- | --- |
| *Ka*POx (2) | x | 350 | 349 | 338 |
| *Sc*POx (3) | 350 | x | 321 | 329 |
| *Ps*POx (4, 5) | 349 | 321 | x | 146 |
| *Mt*CarA (6) | 338 | 329 | 146 | x |

**TmPOx**  **MSTSSSDPFF** **NFAKSSFRSA** **AAQKASASSL** **PPLPGPDKKV** **PGMDIKYDVV** **IVGSGPIGCT** 60

**N1**  **MNDSAA----** **----------** **----------** **----------** **-PVPVEADVL** **VIGSGPVGCT** 25

**N6**  **MSESTA----** **----------** **----------** **----------** **-PAKIRVDVL** **IVGSGPVGCT** 25

**N12**  **MSHQ------** **----------** **----------** **----------** **---KIRVDVL** **IVGSGPVGCT** 21

**N22**  **MSHQ------** **----------** **----------** **----------** **---TIRTDVL** **IVGSGPVGCT** 21

**KaPOx**  **MI--------** **----------** **----------** **----------** **---TRYTDTL** **VVGSGPVGAT** 19

**N29**  **MSHR------** **----------** **----------** **----------** **---TYRTDVL** **IVGSGPVGAT** 21

**N34**  **MSHR------** **----------** **----------** **----------** **---TYRTDVL** **IVGSGPVGAT** 21

**N35**  **MTSHESTSL-** **----------** **----------** **--------QC** **CKAAERTDVL** **IVGSGPVGSA** 31

**ScPOx**  **MTH-------** **----------** **----------** **----------** **---TPRTDVL** **IVGSGIMGSL** 20

**MtCarA** **MSTRVY----** **----------** **----------** **----------** **---PAQVDVA** **IVGSGPAGAT** 23

**PsPOx**  **MSGHRY----** **----------** **----------** **----------** **---PAAVDVA** **IVGSGPTASA** 23

Arm domain

**TmPOx**  **YARELVGA--** **GYKVAMFDIG** **EIDSGLKIGA** **HKKNTV---E** **YQKNIDKFVN** **VIQGQLMSV-** 114

**N1**  **FARKLVEA--** **GKNVLMIDAG** **AQLSR-RYGE** **HLKNSY---L** **FQKNIDLFVS** **VIKGNLLPL-** 78

**N6**  **FARKLVEA--** **GKNVLMIDAG** **AQLSG-RYGE** **HLKNSF---L** **YQRNIDLFVS** **VIRGHLHPL-** 78

**N12**  **FARTLVEA--** **GRSVLMVDAG** **AQLSA-RPGE** **HLKNAF---L** **YQRNVDLFAS** **VIRGHLHPL-** 74

**N22**  **FARTLVEA--** **GRSVLMVDAG** **AQLSA-RPGE** **HLKNAY---L** **YQRNVDLFAS** **VIRGHLHLL-** 74

**KaPOx**  **FARTLVES--** **GREVLMVDAG** **AQLSP-RPGE** **HLKNAY---I** **YQHNTNLFAS** **IIRGHLHLL-** 72

**N29**  **FARTLVDA--** **GRSVLMVEAG** **AQLSA-RPGE** **NLKNAY---I** **YQRDTNLFAS** **VIRGHLHLL-** 74

**N34**  **FARTLVDA--** **GRSVLMVEAG** **AQLSA-RPGE** **NLKNAY---I** **YQRDTNLFAS** **VIRGHLHLL-** 74

**N35**  **FARLIADARP** **SASILMVEAG** **PQLTD-PPGM** **NVRNIPDP-E** **ERAAAQARSQ** **GPNSATAQAG** 89

**ScPOx**  **VARLLRRSDP** **ALHITMADGG** **SPIGG-VPGR** **HLHDLDDPDL** **WSRYNEKVAT** **GIQGMYTGA-** 78

**MtCarA** **YARILSERAS** **SATIAMFEVG** **PTVSD-PPGA** **HVKNIADA-D** **ERAHAQRRSE** **GPHAREDDD-** 80

**PsPOx**  **YARILSEEAP** **GATIAMFEVG** **PTVSN-PPGA** **HVKNIEDP-D** **SRSLAQRASE** **GPGAGAATV-** 80

**TmPOx**  **----------** **---SVPVNTL** **-VVDTLSPTS** **WQAS----TF** **FVRNGSNPEQ** **DPLRNLSGQA** 156

**N1**  **----------** **---STATNRE** **-PVLTLDPSA** **FSYDPDKYAG** **FSMRNQNPEQ** **RGHVNLPAAA** 124

**N6**  **----------** **---SVATNDD** **-PVVTLDPSA** **FRYDPDKYPG** **FVMNNQNPEQ** **KKHDNLGAAA** 124

**N12**  **----------** **---SVPTNDR** **-PEVTLDPSA** **FRVDRDRYRG** **FVRNNQNPDQ** **DRHRNLDAAA** 120

**N22**  **----------** **---SVPTNDR** **-PEVTLDPSA** **FRVDRDRYRG** **FVRNNQNPDQ** **DPHRNLDAAA** 120

**KaPOx**  **----------** **---SVPTSAR** **-AELAVDPAA** **M-AELGSNRS** **SARNAENPDQ** **DPYRNLSAAA** 117

**N29**  **----------** **---SVPTSAR** **-AELAVDPSA** **F-AELGTNRS** **SARNAENPDQ** **DPYRNIPAAA** 119

**N34**  **----------** **---SVPTSAR** **-AELAVDPSA** **F-AELGTNRS** **SARNAENPDQ** **DPYRNIPAAA** 119

**N35**  **EARAVVGVPA** **LGDRPAGAAA** **EGTITARPGT** **FLIDP---GG** **SAADGQPG--** **----AMPAAA** 140

**ScPOx**  **----------** **---EVVRDVA** **GSLPDLTPGM** **FHAL------** **--AFGEDAE-** **----AMPQAA** 112

**MtCarA** **----------** **---RVGGIVK** **SAQRRARPGT** **YLLE----SG** **YQADGED---** **----GLPVAA** 116

**PsPOx**  **----------** **---NSPGAVK** **SGERRARPGT** **YLLQ----DG** **YAFPGED---** **----GMPVAA** 116

**TmPOx**  **VTRVVGGMST** **HWTCATPRFD** **RE-QRP-LL-** **--VKDDADAD** **-DAEWDRLYT** **KAESYFQTGT** 210

**N1**  **ATYAVGGMAT** **HWTCAVPRFH** **PEVERR-YGG** **QG----YPID** **-DKEMDRLYD** **EAESLLARST** 178

**N6**  **ATYAVGGMAT** **HWTCAVPRFH** **PSVERTWNGG** **SGVVHQYPID** **-ATEMDKLYG** **EAEALLNRST** 183

**N12**  **ATYAVGGMAT** **HWTCATPRHH** **PTVERS-DA-** **--------IP** **-AAEWDRLYG** **EAEALLNTRT** 169

**N22**  **ATYAVGGMAT** **HWTCATPRHH** **PVVERS-DA-** **--------IP** **-AAEWDRLYG** **EAERLLNTRT** 169

**KaPOx**  **ACYAVGGMGT** **HWTGATPRHH** **PVLERY-DG-** **--------IS** **-DQEWDGLYG** **EAERLLRVSA** 166

**N29**  **ACYAVGGMAT** **HWTCATPRHH** **PVVERY-NG-** **--------IP** **-AAEWDRCYT** **EAERLLNVSR** 168

**N34**  **ACYAVGGMAT** **HWTCATPRHH** **PVVERY-NG-** **--------IP** **-AAEWDRCYT** **EAERLLNVSR** 168

**N35**  **MSTCVGGMGA** **HWTCATPRPA** **GS-ERI-PF-** **--------IP** **-AAEWDAALD** **EAERLLHVTQ** 188

**ScPOx**  **LAWNAGGMGV** **HWTAATPWPA** **GD-EVF-DF-** **--------GD** **-PDAWAADLD** **TARRLLAVTP** 160

**MtCarA** **FSSNVGGMAA** **HWTGACPRPN** **DS-ERI-GF-** **--------LD** **ETGELDELLS** **EGERLLGVTT** 165

**PsPOx**  **MSSNVGGMAA** **HWTAACPRPG** **GK-ERI-PF-** **--------LP** **---DLEELLN** **DADRLLGVTT** 162

**TmPOx**  **DQFKESIRHN** **LVLNKLTEEY** **KGQ----RDF** **QQIPLAATRR** **SPT----FVE** **WSSANTVFD-** 261

**N1**  **SVFAASARHL** **LVKRVLQRAG** **EEF----ADV** **TELPLAVSDR** **ADSARTSAVT** **WSAADTVLGD** 234

**N6**  **SVFASSARHQ** **LVREVLQRAA** **GEF----YDV** **QELPLAVERR** **ADNARSEMVH** **WSAADTVLGD** 239

**N12**  **DAFDHSVRHQ** **LVREALQREY** **SEL-PEPYEV** **QSLPLAVERR** **TDNPR--MVH** **WSGADTVLGP** 226

**N22**  **DAFDHSVRHR** **LVREALQREY** **SEL-PEPYEV** **QSLPLAVERR** **TDNPR--MVH** **WSGADTVLGP** 226

**KaPOx**  **REFDFSIRQH** **LVTEALRREF** **SEL-PDGYQV** **QSLPLAARRR** **RDNPR--MVH** **WTGVDTVLGD** 223

**N29**  **REFDHSVRQR** **LIIDALREEF** **SEL-PEGYEV** **QSLPLAVKRR** **TDNPR--MVH** **WTGADTVLGD** 225

**N34**  **REFDHSVRQR** **LIIDALREEF** **SEL-PEGYEV** **QSLPLAVKRR** **TDNPR--MVH** **WTGADTVLGD** 225

**N35**  **SAFPDSPQGE** **AIREALREEF** **DGALPEGRKV** **QPLPLAATPR** **PDG----SLR** **WTGTDTVLGP** 244

**ScPOx**  **APIGPTKVGE** **LVLDVLRRRY** **GGTGPADRAP** **QPMPMAVTPT** **PSG----PMP** **RTAPGTIFPP** 216

**MtCarA** **DAFDASPYAG** **IVRERLAAVE** **DAHRDADERV** **QRMPLAVHRR** **DDG----PLV** **WSGSDVVLG-** 220

**PsPOx**  **HAFDGAPFSD** **LVRERLAAVV** **DQGRTPAFRV** **QPMPLAVHRR** **QDG----ALV** **WSGSDVVMG-** 217

**TmPOx**  **LQNRPNTDAP** **EERFNLFPAV** **ACERVVRNAL** **NS---EIESL** **HIHDLIS-GD** **RFEIKADVYV** 317

**N1**  **LA-DPAHTPP** **QGSFTLLPEH** **QCVRLEITGS** **GKHQ-KVQYA** **VVRNLRDVRE** **EIRLRAETYV** 292

**N6**  **LA-DPAHTNS** **QGSFTLLPEH** **QCTRLELTGD** **GKGQTRVEYA** **KVRNLRDLNE** **EIRVNAETYV** 298

**N12**  **LA-DPAHGGA** **Q--FTLLPQH** **LCTRLVLTAD** **GT---RVEYA** **EVRDLND-WR** **TIRVEAETYV** 279

**N22**  **LA-DPASGGA** **Q--FTLLPQH** **LCTRLVLTAD** **GT---RVEYA** **EVRDLND-WR** **TIRVEAENYV** 279

**KaPOx**  **LA-D---GHP** **L--FSLLPQH** **LCTRLVLDRD** **GT---RIAYA** **EVRDLNR-SE** **TVRVVADNYV** 273

**N29**  **LA-DPAGGNA** **R--FTLLPQH** **LCTRLVLDSD** **GT---RIAYA** **EVRDLNG-SR** **TVRVVADHYV** 278

**N34**  **LA-DPAGGNA** **R--FTLLPQH** **LCTRLVLDSD** **GT---RIAYA** **EVRDLNG-SR** **TVRVVADHYV** 278

**N35**  **LADDASSGDP** **R--FTLRPET** **LCRRLVLE-D** **GD---RVTGA** **EVRDLRT-GE** **TSTVRADVVV** 297

**ScPOx**  **LA---QGGDP** **A--FTLLTGT** **LVTALVRD-A** **G----RVTGA** **RLRRVAD-GT** **ESELSADTVV** 265

**MtCarA** **---DITRGNP** **N--FTLFDES** **LVTRVLVE-D** **G----RAAGV** **VVTDVRT-GE** **RRDVRARFVV** 269

**PsPOx**  **---EATRDNP** **Q--FELFDES** **LVTRVLVE-D** **G----TAAGV** **EVQDRRS-GD** **TYQVAARYVV** 266

Head domain

**TmPOx**  **LTAGAVHNTQ** **LLVNSGFGQL** **GRPNPANPPE** **LLPSLGSYIT** **EQSLVFCQTV** **MSTELIDSVK** 377

**N1**  **VACGAVPTPQ** **LLFNSGV---** **-TL-------** **--PALGRYLT** **EQPMSFCQVV** **LQQEHMDHIE** 339

**N6**  **VACGAVLTPQ** **LLYASGI---** **-RL-------** **--PALGRYLT** **EQPMAFCQVV** **LHQEIVDNVE** 345

**N12**  **VACGAVLTPQ** **LLYASGI---** **-RP-------** **--PALGRYLT** **EQPMAFCQVV** **LHQDLVDRVA** 326

**N22**  **VAAGAVLTPQ** **LLYASGI---** **-RP-------** **--PALGRYLT** **EQPMAFCQVV** **LHQDLVDRVA** 326

**KaPOx**  **VAAGAVLAPQ** **LLHASGI---** **-RP-------** **--AALGRYLT** **EHPMAFCQVI** **LLKDLVEQAR** 320

**N29**  **VAAGAVLTPQ** **LLWASGI---** **-RP-------** **--PALGRYLT** **EHPMAFCQVI** **LLQKLVDRAA** 325

**N34**  **VAAGAVLTPQ** **LLWASGI---** **-RP-------** **--PALGRYLT** **EHPMAFCQVI** **LLQKLVDRAA** 325

**N35**  **VAADALRTPQ** **LLWASGI---** **-RP-------** **--PALGRYLT** **EHPLVFSAVA** **LDPELVSAAA** 344

**ScPOx**  **VCADALRTPQ** **LLYASGI---** **-RP-------** **--EALGRHLN** **EHAFVTARVL** **LDLDRFGLDP** 312

**MtCarA** **VAADALRTPQ** **LLWASGI---** **-RP-------** **--DALGRYLN** **DQA----QIV** **FAVRMRDFTP** 312

**PsPOx**  **VGADALRTPQ** **LLWASGI---** **-RP-------** **--DALGRYLN** **DQA----QVV** **FASRLRDVQP** 309

**TmPOx**  **-S------DM** **--TIRGTPGE** **LTYSVTYTPG** **ASTNKHPDWW** **NEKVKNHMM-** **--------QH** 419

**N1**  **-EILRTVPGS** **GDAPAEA---** **----------** **AA--------** **-DRVARYRAA** **QLKRLHSGDK** 376

**N6**  **-TYLRKVPDA** **--RYAEE---** **----------** **AA--------** **-KRVAEYRAE** **QRKKLQEGQP** 380

**N12**  **-T------DP** **--RFAE----** **----------** **----------** **--RVAEHRA-** **--------RH** 342

**N22**  **-T------DP** **--RFAE----** **----------** **----------** **--RVAEHRA-** **--------RH** 342

**KaPOx**  **-T------DQ** **--RFGG----** **----------** **----------** **--QVARHTT-** **--------LF** 336

**N29**  **-T------DP** **--RFAA----** **----------** **----------** **--DVQRHRA-** **--------LF** 341

**N34**  **-T------DP** **--RFAA----** **----------** **----------** **--DVQRHRA-** **--------LF** 341

**N35**  **-A------DA** **--PVSA----** **----------** **----------** **--EAASTAA-** **----------** 358

**ScPOx**  **--------DA** **----------** **----------** **----------** **----------** **----------** 314

**MtCarA** **VV------DA** **--DGVP----** **----------** **----------** **--QT------** **----------** 322

**PsPOx**  **-E------DA** **--PAAA----** **----------** **----------** **--NG------** **----------** 318

Substrate loop

**TmPOx**  **QEDPLPIPFE** **DPEPQVTTLF** **Q-PSHPWHTQ** **IHRDAFSYGA** **VQQSIDS---** **-RLIVDWRFF** 474

**N1**  **TADPVPFPPS** **ERDPNLALLV** **T-EGRPWHCQ** **IHRDAFTYGA** **VPPNVDP---** **-RLIVDLRWF** 431

**N6**  **PADPVPIPKN** **EPEPNLWIPV** **T-EGRPWHCQ** **IHRDAFSYGE** **VPPNVDS---** **-RLIVDLRWF** 435

**N12**  **PEDPVPIPED** **DPEPNVWIPV** **S-EGRPWHCQ** **IHRDAFHYGD** **VPPNVDS---** **-RLIVDLRWF** 397

**N22**  **PEDPVPIPED** **DPEPNVWIPV** **S-EGRPWHCQ** **IHRDAFHYGD** **VPPNVDS---** **-RLIVDLRWF** 397

**KaPOx**  **PDDDLPIPVD** **DPEPNVWIPV** **S-EGRPWHAQ** **ITRDAFHYGD** **VPPHVDG---** **-RLIVDLRWF** 391

**N29**  **PDDALPIPVD** **DPEPNVWIPV** **S-EGRPWHAQ** **ITRDAFHYGD** **VPPHVDG---** **-RLIVDLRWF** 396

**N34**  **PDDALPIPVD** **DPEPNVWIPV** **S-EGRPWHAQ** **ITRDAFHYGD** **VPPHVDG---** **-RLIVDLRWF** 396

**N35**  **------GPAD** **PLTAVVWIPF** **SEEAHPYHAQ** **IMHMATS--P** **FPLDEDSAHA** **GNPVVGMGWF** 410

**ScPOx**  **--LPLPRPGE** **FSTDSLWLPC** **NGPSQPFHGQ** **IMNRTYVDGA** **GRPLAHS---** **----VGLSLY** 365

**MtCarA** **-------GLS** **EYTGVTWVPF** **T-DDMPFHGQ** **VMQLDAS--P** **VKLADDDPAA** **PGSIVGLGLF** 372

**PsPOx**  **-------ALS** **EQSGVAWVPY** **T-DEAPFHGQ** **IMQLDAS--P** **VPLADDDPIV** **PGSIVGLGLF** 368

**TmPOx**  **GRTEPKEENK** **LWFSDKITDA** **YNMPQPTFDF** **RFPAGRTSKE** **AEDMMTDMCV** **MSAKIGGFLP** 534

**N1**  **GISRPRPENK** **VTFSRKIRDT** **FDMPQPTFHF** **CLDE-AERKE** **TDRMNEHMLR** **TAAALGGFMP** 490

**N6**  **GITRPRPENR** **VTFSDTVKDT** **FGMPQPTFHF** **QLSK-QERAE** **AGRMMEHMLR** **VASALGGFMP** 494

**N12**  **GIVEPRPENR** **VTFSDTARDT** **FGMPQPTFEF** **SLSP-QDRAR** **QHRMMADMLR** **AASALGGFLP** 456

**N22**  **GIVEPRPENR** **VTFSDTHRDT** **FGMPQPTFEF** **SLSP-QDRAR** **QHAMMADMLR** **AASALGGFLP** 456

**KaPOx**  **GIVEPRPDNR** **VTFSDTRTDV** **MGMPQPTFEY** **ALSP-QDAER** **QHAMMAEMMR** **AATALGGFLP** 450

**N29**  **GLVDPRPENR** **VTFSDTHTDV** **HGMPQPTFDF** **SFSP-EDAAR** **QHAMMADMLR** **AATALGGFLP** 455

**N34**  **GLVDPRPENR** **VTFSDTHTDV** **HGMPQPTFDF** **SFSD-EDAAR** **QHAMMADMLR** **AATALGGFLP** 455

**N35**  **VRKDPRAEDR** **VTFSDTETDA** **YGMPQMTIHY** **ELTE-RDQAE** **IERAKADMRR** **AAEALGEFVP** 469

**ScPOx**  **VPVESRPQNR** **LVFSPGETDL** **AGLPRIRVEF** **GYSE-TDRAL** **IRRALDEVRS** **VAEEFGPFDP** 424

**MtCarA** **CAKDLQASDR** **VAFSDSDVDG** **YGMPAMQLHY** **TLSD-RDHAT** **IDRAKAEIVR** **LGKAIGDPL-** 430

**PsPOx**  **CAKDLQREDR** **VAFDDDTRDS** **YGLPAMRIHY** **RLTE-RDHVV** **LDRARQEIVR** **LGKAVGEPL-** 426

**TmPOx**  **GSLPQFMEPG** **LVLHLGGTHR** **MGFDEKEDNC** **CVNTDSRVFG** **FKNLFLGGCG** **NIPTAYGANP** 594

**N1**  **GSEPVFLTPG** **LPLHIAGTTR** **MG-TSPLDSV** **V-DEYSKVWN** **IDNLYLGGNG** **LHPFGNASNP** 548

**N6**  **GSEPQFLTPG** **LPLHIAGTTR** **MG-TDAETSV** **V-DRDSKVWG** **IDNLYLGGNG** **LHPFGNAANP** 552

**N12**  **GSEPRFVAPG** **LPLHIAGTTR** **MG-TDAETSV** **V-DTDSRVWG** **IDNLYLGGNG** **LIPTGNASNP** 514

**N22**  **GSEPRFVAPG** **LPLHIAGTTR** **MG-TDPETSV** **V-DTDSRVWG** **IDNLYLGGNG** **VIPTGNASNP** 514

**KaPOx**  **GSEPRFTAPG** **LPLHIAGTIR** **MG-DDPQSSV** **V-DTDSRVWG** **LENLYLGGNG** **VIPTGTACNP** 508

**N29**  **GAEPRFTTPG** **LPLHIAGTVR** **MG-TDPQTSV** **V-DTDSRVWG** **FENLYLGGNG** **VIPTATASNP** 513

**N34**  **GAEPRFTTPG** **LPLHIAGTVR** **MG-TDPQTSV** **V-DTDSRVWG** **FENLYLGGNG** **VIPTATASNP** 513

**N35**  **GGEPRLMPAG** **SSLHYQGTVR** **MGPADDGTSV** **C-DPHSRVWG** **FENLYLGGNG** **VIPTATACNP** 528

**ScPOx**  **ATESTVLPPG** **SSLHLTGTVR** **AGVTDDGTGV** **C-DPDGRVWG** **FDNLYLAGNG** **VVPTPMAANV** 483

**MtCarA** **DDRPFVMPLG** **ASLHYQGTVR** **MGLADDGASV** **C-SPDSEVWG** **APGLFVAGNG** **VIPTATACNP** 489

**PsPOx**  **DERPFVLPPG** **ASLHYQGTTR** **MGETDDGESV** **C-SPDSQVWQ** **VPGLFVAGNG** **VIPTATACNP** 485

**TmPOx**  **TLTAMSLAIK** **SCEYIKQNFT** **PSPFTSEAQ-** **---------** 623

**N1**  **TLTSVATALH** **AADTIVKGHP** **GH--------** **---------** 570

**N6**  **TLTSVAMALH** **AAESILSNHP** **SS--------** **---------** 574

**N12**  **TLTSVAMALR** **AARHILGRAP** **ATTAKRRAAD** **GPGLLAVR-** 552

**N22**  **TLTSVAMALR** **AARHILGRAP** **AT--------** **---------** 536

**KaPOx**  **TLTSVAMALK** **AAHHLAGSRE** **ARERRRTGAD** **--EVLAVRS** 545

**N29**  **TLTSVAMALK** **AAHHLVGRAQ** **ATEARRPG--** **---VLAVR-** 546

**N34**  **TLTSVAMALK** **AAHHLVGRLQ** **ATEARRPG--** **---VLAVR-** 546

**N35**  **TLTSVALAVR** **AARALVAR--** **----------** **---------** 546

**ScPOx**  **TLTGAVTAVR** **TARAVTART-** **----------** **---------** 502

**MtCarA** **TLTGVALAVR** **GARHIADEIT** **ADLA------** **---------** 513

**PsPOx**  **TLTSVALAVR** **GARKIAEEIT** **SSLLMSESDN** **---RLSK--** 519

**Figure S8.** Multiple sequence alignment of the sequences for fungal *Tm*POx (1) and bacterial *Ka*POx (2), *Ps*POx (4, 5), *Sc*POx (3) and *Mt*CarA (6), together with ancestral sequences of N1, N6, N12, N22, N29, N34 and N35. Important structural and catalytical features are framed in black dotted or pink boxes.

**Table S6.** Amino acid sequences (N- to C-terminus) of the target ancestors.

| **Ancestor** | **Amino acid sequence** |
| --- | --- |
| **N1** | MNDSAAPVPVEADVLVIGSGPVGCTFARKLVEAGKNVLMIDAGAQLSRRYGEHLKNSYLFQKNIDLFVSVIKGNLLPLSTATNREPVLTLDPSAFSYDPDKYAGFSMRNQNPEQRGHVNLPAAAATYAVGGMATHWTCAVPRFHPEVERRYGGQGYPIDDKEMDRLYDEAESLLARSTSVFAASARHLLVKRVLQRAGEEFADVTELPLAVSDRADSARTSAVTWSAADTVLGDLADPAHTPPQGSFTLLPEHQCVRLEITGSGKHQKVQYAVVRNLRDVREEIRLRAETYVVACGAVPTPQLLFNSGVTLPALGRYLTEQPMSFCQVVLQQEHMDHIEEILRTVPGSGDAPAEAAADRVARYRAAQLKRLHSGDKTADPVPFPPSERDPNLALLVTEGRPWHCQIHRDAFTYGAVPPNVDPRLIVDLRWFGISRPRPENKVTFSRKIRDTFDMPQPTFHFCLDEAERKETDRMNEHMLRTAAALGGFMPGSEPVFLTPGLPLHIAGTTRMGTSPLDSVVDEYSKVWNIDNLYLGGNGLHPFGNASNPTLTSVATALHAADTIVKGHPGH |
| **N6** | MSESTAPAKIRVDVLIVGSGPVGCTFARKLVEAGKNVLMIDAGAQLSGRYGEHLKNSFLYQRNIDLFVSVIRGHLHPLSVATNDDPVVTLDPSAFRYDPDKYPGFVMNNQNPEQKKHDNLGAAAATYAVGGMATHWTCAVPRFHPSVERTWNGGSGVVHQYPIDATEMDKLYGEAEALLNRSTSVFASSARHQLVREVLQRAAGEFYDVQELPLAVERRADNARSEMVHWSAADTVLGDLADPAHTNSQGSFTLLPEHQCTRLELTGDGKGQTRVEYAKVRNLRDLNEEIRVNAETYVVACGAVLTPQLLYASGIRLPALGRYLTEQPMAFCQVVLHQEIVDNVETYLRKVPDARYAEEAAKRVAEYRAEQRKKLQEGQPPADPVPIPKNEPEPNLWIPVTEGRPWHCQIHRDAFSYGEVPPNVDSRLIVDLRWFGITRPRPENRVTFSDTVKDTFGMPQPTFHFQLSKQERAEAGRMMEHMLRVASALGGFMPGSEPQFLTPGLPLHIAGTTRMGTDAETSVVDRDSKVWGIDNLYLGGNGLHPFGNAANPTLTSVAMALHAAESILSNHPSS |
| **N12** | MSHQKIRVDVLIVGSGPVGCTFARTLVEAGRSVLMVDAGAQLSARPGEHLKNAFLYQRNVDLFASVIRGHLHPLSVPTNDRPEVTLDPSAFRVDRDRYRGFVRNNQNPDQDRHRNLDAAAATYAVGGMATHWTCATPRHHPTVERSDAIPAAEWDRLYGEAEALLNTRTDAFDHSVRHQLVREALQREYSELPEPYEVQSLPLAVERRTDNPRMVHWSGADTVLGPLADPAHGGAQFTLLPQHLCTRLVLTADGTRVEYAEVRDLNDWRTIRVEAETYVVACGAVLTPQLLYASGIRPPALGRYLTEQPMAFCQVVLHQDLVDRVATDPRFAERVAEHRARHPEDPVPIPEDDPEPNVWIPVSEGRPWHCQIHRDAFHYGDVPPNVDSRLIVDLRWFGIVEPRPENRVTFSDTARDTFGMPQPTFEFSLSPQDRARQHRMMADMLRAASALGGFLPGSEPRFVAPGLPLHIAGTTRMGTDAETSVVDTDSRVWGIDNLYLGGNGLIPTGNASNPTLTSVAMALRAARHILGRAPATTAKRRAADGPGLLAVR |
| **N22** | MSHQTIRTDVLIVGSGPVGCTFARTLVEAGRSVLMVDAGAQLSARPGEHLKNAYLYQRNVDLFASVIRGHLHLLSVPTNDRPEVTLDPSAFRVDRDRYRGFVRNNQNPDQDPHRNLDAAAATYAVGGMATHWTCATPRHHPVVERSDAIPAAEWDRLYGEAERLLNTRTDAFDHSVRHRLVREALQREYSELPEPYEVQSLPLAVERRTDNPRMVHWSGADTVLGPLADPASGGAQFTLLPQHLCTRLVLTADGTRVEYAEVRDLNDWRTIRVEAENYVVAAGAVLTPQLLYASGIRPPALGRYLTEQPMAFCQVVLHQDLVDRVATDPRFAERVAEHRARHPEDPVPIPEDDPEPNVWIPVSEGRPWHCQIHRDAFHYGDVPPNVDSRLIVDLRWFGIVEPRPENRVTFSDTHRDTFGMPQPTFEFSLSPQDRARQHAMMADMLRAASALGGFLPGSEPRFVAPGLPLHIAGTTRMGTDPETSVVDTDSRVWGIDNLYLGGNGVIPTGNASNPTLTSVAMALRAARHILGRAPAT |
| **N29** | MSHRTYRTDVLIVGSGPVGATFARTLVDAGRSVLMVEAGAQLSARPGENLKNAYIYQRDTNLFASVIRGHLHLLSVPTSARAELAVDPSAFAELGTNRSSARNAENPDQDPYRNIPAAAACYAVGGMATHWTCATPRHHPVVERYNGIPAAEWDRCYTEAERLLNVSRREFDHSVRQRLIIDALREEFSELPEGYEVQSLPLAVKRRTDNPRMVHWTGADTVLGDLADPAGGNARFTLLPQHLCTRLVLDSDGTRIAYAEVRDLNGSRTVRVVADHYVVAAGAVLTPQLLWASGIRPPALGRYLTEHPMAFCQVILLQKLVDRAATDPRFAADVQRHRALFPDDALPIPVDDPEPNVWIPVSEGRPWHAQITRDAFHYGDVPPHVDGRLIVDLRWFGLVDPRPENRVTFSDTHTDVHGMPQPTFDFSFSPEDAARQHAMMADMLRAATALGGFLPGAEPRFTTPGLPLHIAGTVRMGTDPQTSVVDTDSRVWGFENLYLGGNGVIPTATASNPTLTSVAMALKAAHHLVGRAQATEARRPGVLAVR |
| **N34** | MSHRTYRTDVLIVGSGPVGATFARTLVDAGRSVLMVEAGAQLSARPGENLKNAYIYQRDTNLFASVIRGHLHLLSVPTSARAELAVDPSAFAELGTNRSSARNAENPDQDPYRNIPAAAACYAVGGMATHWTCATPRHHPVVERYNGIPAAEWDRCYTEAERLLNVSRREFDHSVRQRLIIDALREEFSELPEGYEVQSLPLAVKRRTDNPRMVHWTGADTVLGDLADPAGGNARFTLLPQHLCTRLVLDSDGTRIAYAEVRDLNGSRTVRVVADHYVVAAGAVLTPQLLWASGIRPPALGRYLTEHPMAFCQVILLQKLVDRAATDPRFAADVQRHRALFPDDALPIPVDDPEPNVWIPVSEGRPWHAQITRDAFHYGDVPPHVDGRLIVDLRWFGLVDPRPENRVTFSDTHTDVHGMPQPTFDFSFSDEDAARQHAMMADMLRAATALGGFLPGAEPRFTTPGLPLHIAGTVRMGTDPQTSVVDTDSRVWGFENLYLGGNGVIPTATASNPTLTSVAMALKAAHHLVGRLQATEARRPGVLAVR |
| **N35** | MTSHESTSLQCCKAAERTDVLIVGSGPVGSAFARLIADARPSASILMVEAGPQLTDPPGMNVRNIPDPEERAAAQARSQGPNSATAQAGEARAVVGVPALGDRPAGAAAEGTITARPGTFLIDPGGSAADGQPGAMPAAAMSTCVGGMGAHWTCATPRPAGSERIPFIPAAEWDAALDEAERLLHVTQSAFPDSPQGEAIREALREEFDGALPEGRKVQPLPLAATPRPDGSLRWTGTDTVLGPLADDASSGDPRFTLRPETLCRRLVLEDGDRVTGAEVRDLRTGETSTVRADVVVVAADALRTPQLLWASGIRPPALGRYLTEHPLVFSAVALDPELVSAAAADAPVSAEAASTAAGPADPLTAVVWIPFSEEAHPYHAQIMHMATSPFPLDEDSAHAGNPVVGMGWFVRKDPRAEDRVTFSDTETDAYGMPQMTIHYELTERDQAEIERAKADMRRAAEALGEFVPGGEPRLMPAGSSLHYQGTVRMGPADDGTSVCDPHSRVWGFENLYLGGNGVIPTATACNPTLTSVALAVRAARALVAR |

| **N67** | MSHNPTIAIVGSGPIGSAYARLLLESLPDARVVMFEAGPQLTDRPGESVRNIADPDEKARAREMSQGPQAGAFRESLGIPAGTVVEGMFTARQGTHLLDFGGEGSAHAPTFPAAAAATNVGGQGAHWTCATPRPAFSEKIPFIADDEWDDLIATAEGLLHVQSAAFADSAVGGAIRSLLEEEFGAELPEGYGPSTLPVAGDPQPDGSMRWAGADVVLGPLIDPGSPLSERFELRDLTLVRRVEHDGDRVTGVTVEDLRTRETSFVPADLVVVAADAFRSPQLLWASGIRPRALGHYLTEHPVVISTVALDAERMSRFATEEDLDAELARRATNPADPVAAVNRIPFSEPDHPFSLQVMYSETTPFPMDPDAPHANNRWGYVNMGYGMRKHPRFEDAVTFDDDEPDYRGFPNMTIEYALTEAEQAEIAEATERLRRAGNALGTFVAEPRLMPNGSSLHYQGTMRMGEADDGTSVADPWSRVWGYENLVVGGNALIPTATAMNPTLMSVAIAVRGARKAAEE |
| --- | --- |
| **N167** | MTSTQSTADVLIVGSGIMGAAVARQVREARPGARILMVDAGPVIGSVPGQHLHDSPDEEIWARYNQRVASGVQSMYVGAATTADVGGSLASVEPGMYHLSALGEDAAAMPAAALAWNVGGMGVHWTAACPWPWGSEVFDFIPAAEWDADLDTAQRLLRVHPSPFGPTEAGDAVLEALDEVFGPVSAPGRHPQPMPMAVTPSPSGPLRRTGPNRIFPPIADGGDPAFTLLPGTLCTRLLHDGGRVTGARVRDIATGEERTIEASVVVVCADTLRTPQLLFASGIRPPALGRHLNEHAFLTGRVLADLERLGVDLSSLPAPREGEWAVGSYWLPHSGAAQPFHGQIMDRPFVDEDGEPLAYSVGLSLYVPTEVRAENRLEFSDTETDAAGMPRMTVHFSYSDRDLALIERARAQQRQAAEALGDFDPERDSALLPPGSSLHYTGTVRMGPADDGTSVCDPDGRVWGFDNLYLAGNGVIPTPVVCNSTLTGTVTAVRAARAVAAR |
| **N202** | MTDTPRTDVLIVGSGIMGSVVARLLRETDPALRITMVDGGSAIGSAPGLHLHDVDDPVLWSRYNEQVGTGIQGMYTGAEVVREVADSLTGLTPGMFHALAFGEDAEAMPATALAWNAGGMGVHWTAATPWPAGDEVFDFGDPARWAADLDTARRLLAVTPAAIGPTEVGRLVLDVLRRRYDGVGPDDRRPQPMPMAVTATASGPMPRTAPGTIFPAIATGGDPAFTLLTGTLATSLLVSGGRVTGARLRRVADGAESELRADTVVVCADALRTPQLLFASGIRPEALGRYLNEHAFITARVLLDLDRFGIGLDALPLPRVGEFSTDSLWLPQNGAAQPFHGQIMNRTYVDEDGRPLAHSVGLSLYVPVESRPENRLVFSETETDLAGMPRIGVEFGYSDADRALIGRALDEVRSLAEEFGPFDPATERALLPPGSSLHLTGTVRAGAADDGTSVCDPDGRVWGFDNLYLAGNGVIPTAMAANVTLTGAVTAVRAARAVAAR |
| **N284** | MTTEYDVVVVGSGPVGSAFARRVHDAAPSARVLMVEAGPRLTDPPGAHVRNLPDAEERAAAQARSQGPNPATAQVSAASATVSAPAPVGASREGAVTARPGTFLIRPGSAADGQTGMPAAAMSSNVGGMGAHWTCACPRPGDSERIPFIPAAELDAALAEAERLLHVTQDAFPDTPLGDEVRRALGEVFDEGRPPDRRVQPMPLAVTPRPDGSPRWSGTDTVLGDAASSDPRFELRPETLCRRVLLEDGRVTGVELRDLRTGEVHTVRARAVVVAADALRTPQLLWASGIRPPALGRYLNDHPQVVSAVRLDDELVAAPAAHAETSADGARDLLTGVSWVPFSDERHPFHGQVMQLDASPVPLGDDDSADPGSVVGLGWFAAKDIRAEDRVEFSDTETDAYGMPAMTIHYSLTDRDRATIERAKEAVTRAAAALGEPLPGEPRLLPAGSSLHYQGTVRMGPADDGTSVCDPHSRVWGVENLYVGGNGVIPTATACNPTLTSVALAVRAARAIAAR |
| **N327** | MTRRYPSSVDVAIVGSGPAGAAYARILSEQAPEATIAVFEVGPTVSDPPGAHVKNIADPAERARAQRRSEGPSAASPTVTNPGAAKDGARRARPGTFLLPDGWRVEGEDGLPAAAMSSNVGGMGAHWTGACPRPGGSERIAFLPDLDELLDEAERLLGVSADAFADAPFADEVRARLGAALDEGRAPDRRVQPMPLAVHRRDDGRLVWSGSDVVFGDATRANPNFTLFDESLVTRVLVEDGRAAGVRVRDLRTGEEHEVRARFVVVAADALRTPQLLWASGIRPPALGRYLNDQPQVVFAVRLRDVAPAPASAPAAAADSSADGALTAQSGVSWVPFTDEHPFHGQVMQLDASPVPLADDDDPAPGSIVGLGWFCAKDLQASDRVEFSDDETDAYGMPAMRIHYTLTERDHETIARAREAIVRAAKALGEPLDDEPLTLPPGSSLHYQGTVRMGETDDGTSVCDPDSQVWGVPGLYVAGNGVIPTATACNPTLTSVALAVRGARAIAAE |
| **N383** | MSARRYPASVDVAIVGSGPTGAAYARILSEQAPDATIAMFEVGPTVSDPPGAHVKNIADPAARAAAQRRSEGPGAGAATVSSPGAVKSGQRRARPGTFLLEDGYQVDGEDGLPVAAMSSNVGGMAAHWTGACPRPGGSERIAFLPDLDELLDEAERLLGVTTDAFDGAPFGDLVRERLAAAVDEGREPETRVQRMPLAVHRRDDGRLVWSGSDVVLGDVTRANPNFTLFDESLVTRVLVEDGRAAGVEVRDRRTGETHTVRARFVVVAADALRTPQLLWASGIRPDALGRYLNDQAQVVFATRIRDVAPAAAADAAAAGALSEQSGVSWVPFTDEMPFHGQIMQLDASPVPLAEDDPVVPGSIVGLGLFCAKDLQRDDRVEFSDDEVDAYGMPAMRIHYRLTDRDHEVIDRAREEIVRLGKAVGEPLDDRPFTMPLGASLHYQGTTRMGETDDGESVCSPDSEVWGVPGLFVAGNGVIPTATACNPTLTSVALAVRGARRIAAE |

**References**

1. Leitner C, Volc J, Haltrich D. 2001. Purification and Characterization of Pyranose Oxidase from the White Rot Fungus *Trametes multicolor*. Appl Environ Microbiol 67:3636–3644.

2. Herzog PL, Sützl L, Eisenhut B, Maresch D, Haltrich D, Obinger C, Peterbauer CK. 2019. Versatile oxidase and dehydrogenase activities of bacterial pyranose 2-oxidase facilitate redox cycling with manganese peroxidase *in vitro*. Appl Environ Microbiol 85:1–15.

3. Kostelac A, Sützl L, Puc J, Furlanetto V, Divne C, Haltrich D. 2022. Biochemical Characterization of Pyranose Oxidase from *Streptomyces canus*—Towards a Better Understanding of Pyranose Oxidase Homologues in Bacteria. Int J Mol Sci 23:1–15.

4. Mendes S, Banha C, Madeira J, Santos D, Miranda V, Manzanera M, Ventura MR, van Berkel WJH, Martins LO. 2016. Characterization of a bacterial pyranose 2-oxidase from *Arthrobacter siccitolerans*. J Mol Catal B Enzym 133:S34–S43.

5. Taborda A, Frazão T, Rodrigues M V., Fernández-Luengo X, Sancho F, Lucas3 MF, Frazão C, Ventura MR, Masgrau L, Borges PT, Martins LO. 2023. Mechanistic Insights into Glycoside 3-Oxidases Involved in C-Glycoside Metabolism in Soil Microorganisms. Prepr (Version 1) available Res Sq https://doi.org/10.21203/rs.3.rs-2662172/v1.

6. Kumano T, Hori S, Watanabe S, Terashita Y, Yu HY, Hashimoto Y, Senda T, Senda M, Kobayashi M. 2021. FAD-dependent C -glycoside–metabolizing enzymes in microorganisms: Screening, characterization, and crystal structure analysis. Proc Natl Acad Sci 118.

7. Studer G, Rempfer C, Waterhouse AM, Gumienny R, Haas J, Schwede T. 2020. QMEANDisCo—distance constraints applied on model quality estimation. Bioinformatics 36:1765–1771.

8. Daniel G, Volc J, Kubatova E. 1994. Pyranose oxidase, a major source of H2O2 during wood degradation by Phanerochaete chrysosporium, trametes versicolor, and Oudemansiella mucida. Appl Environ Microbiol 60:2524–2532.
